# Recent Advances in the Synthesis of Borinic Acid Derivatives

**DOI:** 10.3390/molecules28062660

**Published:** 2023-03-15

**Authors:** Marion Boyet, Laurent Chabaud, Mathieu Pucheault

**Affiliations:** Institut des Sciences Moléculaires, University Bordeaux, CNRS, Bordeaux INP, ISM, UMR 5255, F-33400 Talence, France

**Keywords:** borinic acids, heterocycles, organoboron compounds, synthetic approaches, tetracoordinated

## Abstract

Borinic acids [R_2_B(OH)] and their chelate derivatives are a subclass of organoborane compounds used in cross-coupling reactions, catalysis, medicinal chemistry, polymer or optoelectronics materials. In this paper, we review the recent advances in the synthesis of diarylborinic acids and their four-coordinated analogs. The main strategies to build up borinic acids rely either on the addition of organometallic reagents to boranes (B(OR)_3_, BX_3_, aminoborane, arylboronic esters) or the reaction of triarylboranes with a ligand (diol, amino alcohol, etc.). After general practical considerations of borinic acids, an overview of the main synthetic methods, their scope and limitations is provided. We also discuss some mechanistic aspects.

## 1. Introduction

Organoboron compounds are considered as a mainstay in modern organic chemistry for the construction of carbon–carbon (Suzuki–Miyaura cross-coupling, Petasis reaction, etc.) or carbon–heteroatom bonds (Chan–Lam–Evans coupling, oxidation, etc.), but also in numerous transversal fields including catalysis, materials science, biology, imaging, etc. [1,2]. Among boron derivatives, borinic acids [R_2_B(OH)] are far less studied than their parent boronic acids [RB(OH)_2_], although they display interesting properties and reactivities. Borinic acids contain two C–B bonds and one B–O bond, resulting in an enhanced Lewis acidity in comparison to boronic acids. From a structural point of view, it has been shown that borinic acids can exist as a monomer, dimer (anhydride R_2_BOBR_2_), or cyclic trimer ([R_2_BOH]_3_), in solution or the solid state, depending on the substitution pattern of the R group [3,4,5].

As well as some applications of borinic acids in cross-coupling reactions [6,7], or as bioactive compounds [8,9,10], they have mainly been used for their propensity to coordinate alcohols, diols, amino alcohols, etc. [11]. For instance, borinic acids catalyze regioselective functionalization of diols, carbohydrates or epoxide ring opening reactions [12,13]. Four-coordinated compounds derived from borinic acids have also been intensively studied in optoelectronics including OLEDs [14].

**Figure 1:**
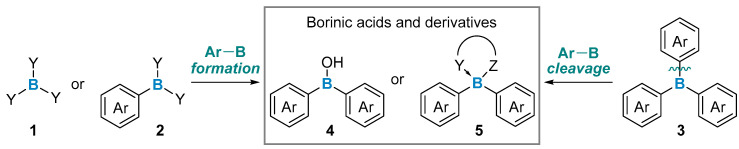
General methods for the synthesis of borinic acids **4** and their derivatives **5**.

Borinic acids and their derivatives (borinic esters [R_2_B(OR’)] or four-coordinated borinic acids) are prepared by three main methods that we sorted depending on the number of C–B bonds formed or cleaved, and the starting borylating reagents. The first one relies on the formation of two carbon–boron bonds using borylating agents **1** such as trialkoxyboranes, borontrihalides, etc. (Figure 1). The second approach starts from arylboron reagents **2**, and requests the formation of one carbon–boron bond to access borinic acids **4**. Finally, the last method involves the cleavage of a C–B bond of triarylboranes **3**, either by hydrolysis to produce **4**, or by reaction with a bidentate ligand (amino alcohol, diamine, etc.) to give the corresponding four-coordinated borinic derivatives **5**.

**Scheme 1:**
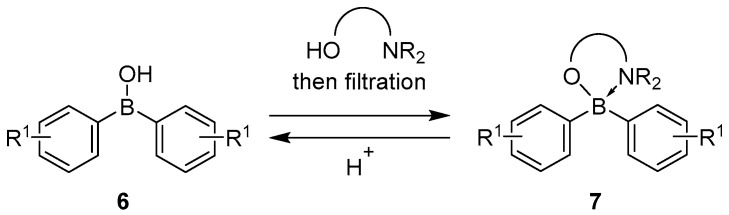
Synthesis of amino alcohol borinate complexes **7**.

Due to their high Lewis acidity, borinic acids are more prone to oxidation (by oxygen from the air) and protodeboronation (under acidic conditions) than their parent boronic acids. Accordingly, purification over silica gel generally results in a low isolated yield. An alternative way of purifying borinic acids is to form a chelate complex with amino alcohols, typically, ethanolamine [15]. The resulting stable aminoborinates **7** usually precipitate and can be filtered off to remove the soluble impurities (boronic acid derivatives, etc.) (Scheme 1). As a result of the coordination of the nitrogen lone pair to the boron atom, which prevents oxidation and protodeboronation, aminoborinates **7** can be easily stored at room temperature without any precaution. Interestingly, the free borinic acids **6** can be released by a simple treatment under smooth acidic conditions.

## 2. Formation of Two Carbon–Boron Bonds

### 2.1. By Nucleophilic Addition of ArLi and ArMgX to Boron Reagents

The nucleophilic addition of two equivalents of an organolithium or organomagnesium reagent onto an electrophilic boron species (such as trialkoxyboranes, trihalogenoboranes or aminoboranes) is a straightforward method to synthesize symmetric diarylborinic acids. This strategy allows for the preparation of both acyclic and cyclic borinic acids, using cheap and readily accessible borylating agents. One major drawback of this strategy is the potential formation of undesired side products such as boronic acids and triarylboranes, resulting in difficult purifications.

#### 2.1.1. Use of Trialkoxyboranes

**Scheme 2:**
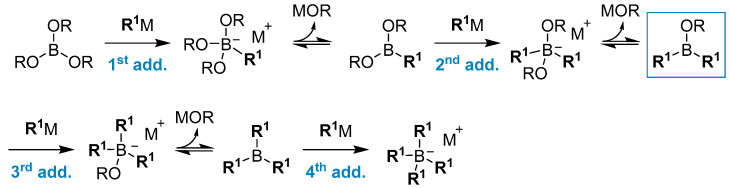
Addition of organometallic species onto trialkoxyboranes.

Organoboron compounds, including boronic acids, borinic acids and borane derivatives can be prepared by the addition of an organometallic species onto trialkoxyborane reagents. Although the synthesis of boronic acid derivatives usually performs well, with high yield and purity using an excess of trialkoxyboranes, the scenario becomes more complex with borinic acids (Scheme 2). In this case two equivalents of an organometallic reagent RM (M = Li, MgX) are needed: the first one reacts with the trialkoxyborane to provide the boronic ester after metal alkoxide elimination [16], while the second addition gives rise to desired borinic ester. However, borinic esters are more electrophilic than their parent boronic esters. Thus, depending on the reaction conditions (stoichiometry, temperature or rate of addition of the organometallic), formation of triarylboranes and tetraarylborates can then arise from a third and fourth addition, respectively.

The uncontrolled polyaddition can be sometimes avoided, or at least limited by careful optimization of the reaction conditions. Along with the reaction temperature and the organometallic species, the nature of the borylating agent plays a crucial role. It appears that lower yields are usually obtained with trimethyl borate (B(OMe)_3_) [17] compared to B(O*n*-Bu)_3_ [7] and B(O*i*-Pr)_3_ [18]. This drop in isolated yield can be attributed to the formation of a substantial amount of boronic acid [19], but also to the quality of the borylating agent. Indeed, B(OMe)_3_ is highly moisture sensitive, and even the commercial source can contain a small amount of MeOH, which significantly affects the overall yield, by hydrolyzing the organometallic reagent. Other trialkoxyboranes such as B(O*t*-Bu)_3_ [20], PinBO*i*-Pr [21,22], and butylboroxine [23], have been sporadically studied for the synthesis of borinic acid derivatives.

**Scheme 3:**
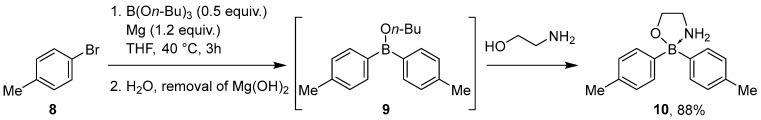
Synthesis of borinic acid ethanolamine complex **10**.

A recent study by Zou et al. also showed the importance of the workup conditions (Scheme 3) [15]. As just mentioned, they observed the selective formation of the *p*-tolylborinic ester **9** when using tributyl borate B(O*n*-Bu)_3_, instead of B(OMe)_3_ or B(O*i*-Pr)_3_. In their previous procedures, aqueous hydrochloric acid was used to hydrolyze the intermediate borinic ester **9** and solubilize the magnesium salts (MgX_2_, MgXOH).

Interestingly, they found that simply adding water instead of 1 M HCl (aq) resulted in the precipitation of Mg(OH)_2_ that could be easily removed by filtration. Subsequent reaction of the borinic ester with ethanolamine afforded **10** with an excellent 88% yield. This method clearly simplifies the procedure with Grignard reagents and could be used with other amino alcohols, or amino acids.

**Scheme 4:**
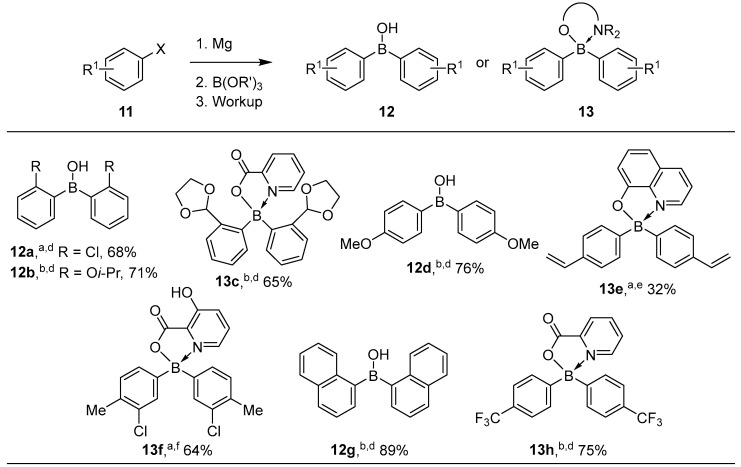
Synthesis of substituted diarylborinic acids and derivatives. (a) B(OMe)_3_ was used. (b) B(O*n*-Bu)_3_ was used. (c) B(O*i*-Pr)_3_ was used. (d) X = Br, (e) X = Cl, (f) X = I.

Regarding the substituent on the aromatic ring, these methods allow the synthesis of a variety of *ortho-*, *meta-* and *para-*substituted borinic acids **12** or their amino alcohol derivatives **13** as exemplified in Scheme 4.

In general, good yields are obtained whatever the substitution pattern on the aromatic ring. Furthermore, electron-withdrawing (i.e., **12a** [24], antibacterial agent **13f [25]**, **13h [15]**) and -donating (**12b** [6,26], **12d**) [7] substituents are tolerated, as well as napththyl groups (i.e., **12g**, a 1,2-diol complexing agent for absolute configuration prediction) [27]. However, the reaction is less efficient for *para* vinyl-substituted borinic acid **13e** [28], probably because of the potential reaction of the styrenyl moiety with the Grignard reagent. This is a main drawback of this strategy because only functional groups compatible with Grignard reagents (or organolithium) and acidic workup are allowed, which considerably narrows the scope of the reaction. However, the recent method reported by Zou et al. using water instead of HCl for the hydrolysis, enables the synthesis of diketal **13c** in a good 65% yield [15].

**Scheme 5:**
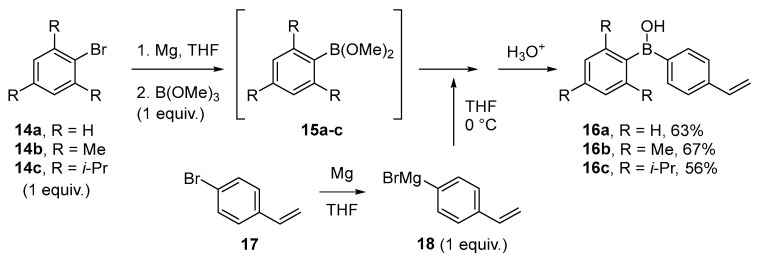
Synthesis of polymerizable borinic acids **16a–c**.

Non-symmetrical borinic acids are of particular interest for applications in medicinal chemistry or materials science. For instance, in the course of their study on borinic-acid-containing polymers as chemical sensors, Wan et al. used a one-pot synthesis of monomers **16a–c** by the sequential addition of Grignard reagent on trimethoxyborane (Scheme 5) [29,30]. The reaction of the Grignard of **14** with B(OMe)_3_ produced boronic ester **15**, which was in situ reacted with the other Grignard (i.e., **18**) derived from *p*-bromostyrene. By careful control of the temperature and the stoichiometry, polymerizable borinic acids **16a–c** were selectively obtained in good yields after purification on column chromatography.

**Scheme 6:**
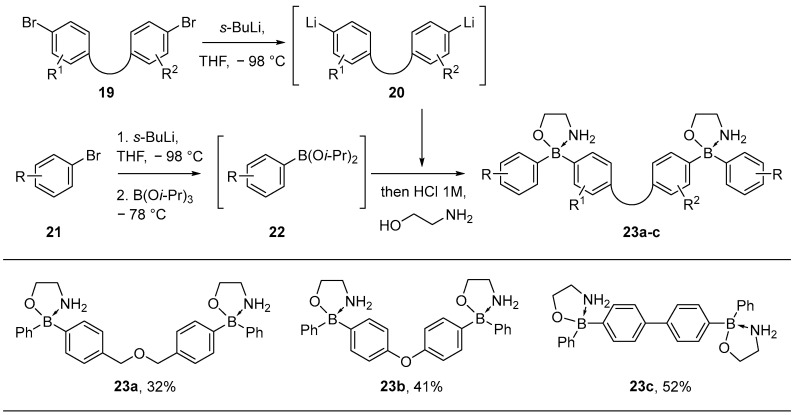
Synthesis of diamino borinates **23a–c** by in situ generation of borinic esters.

Such a strategy has also been exploited with aryllithium species for the synthesis of diborinic acid derivatives **23** that display strong inhibitory activity on store-operated calcium entry[10]. Starting from commercially available arylbromides **21**, lithiation at a low temperature, then borylation with B(O*i*-Pr)_3_, afforded intermediates **22**, ready to react with preformed bis-phenyllithium **20** (Scheme 6). Hydrolysis, then esterification with ethanol amine afforded a range of bis-borinic acid derivatives **23** tethered by a biphenyl or an ether linker.

**Scheme 7:**
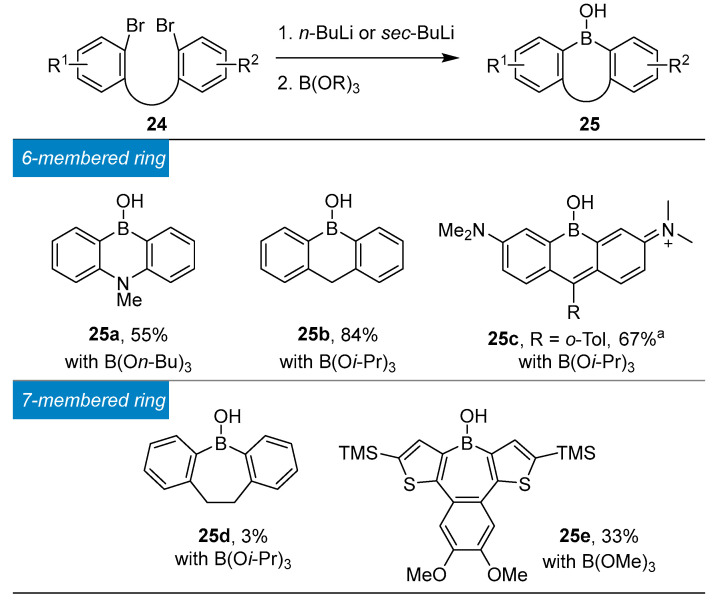
Synthesis of cyclic borinic acids **25**. (a) Obtained after treatment with chloranil, then 6N HCl.

Cyclic analogues of borinic acids are an important subclass of B-containing heterocycles. They have been used for several applications such as catalysts for regioselective activation of polyols or epoxide opening [31], building blocks in double Suzuki coupling [32,33], sensors [34] or fluorescent emitters [35]. A straightforward method to access these heterocycles relies on the reaction of an electrophilic boron reagent with a diaryllithium species, which can either be generated by halide–lithium exchange or by directed *ortho*-metalation. As described in Scheme 7, the halide–lithium exchange strategy has been successfully applied to the synthesis of various six-membered B-heterocycles such as azaborine **25a** (used for catalysis) [36,37], methylene-bridged diarylborinic acid **25b** [32], or fluorescent borinic acid **25c** [34], a ratiometric sensor for H_2_O_2_. All these compounds were isolated in moderate to good yields whatever the source of borate reagent (B(O*n*-Bu)_3_ or B(O*i*-Pr)_3_). Borepinol derivatives **25d–e** that embedded a seven-membered ring were also prepared by this strategy, albeit with more modest yields. For example, compound **25d [8]** was isolated in a low 3% yield, which was attributed by the authors to the tedious purification and extensive decomposition of **25d** over silica gel. In contrast, the aromaticity of borepinol **25e** confers to the molecule stability towards air and moisture, and could be isolated with a 33% yield [35]. Interestingly, this compound proved to be an efficient probe to selectively bind dopamine over norepinephrine.

**Scheme 8:**
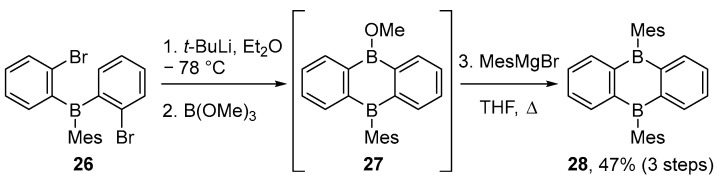
Synthesis of intermediate borinic ester **27**.

A related approach was described for the synthesis of 9,10-diboraanthracene **28** (a fluoride ion sensor) from triarylborane **26** (Scheme 8) [38]. Dilithiation of **26** with *t*-BuLi, followed by addition of trimethyl borate led to borinic ester intermediate **27**. In this specific example, compound **27** was not hydrolyzed but was directly reacted with mesityl magnesium bromide to afford **28** with a 47% yield over three steps.

**Scheme 9:**
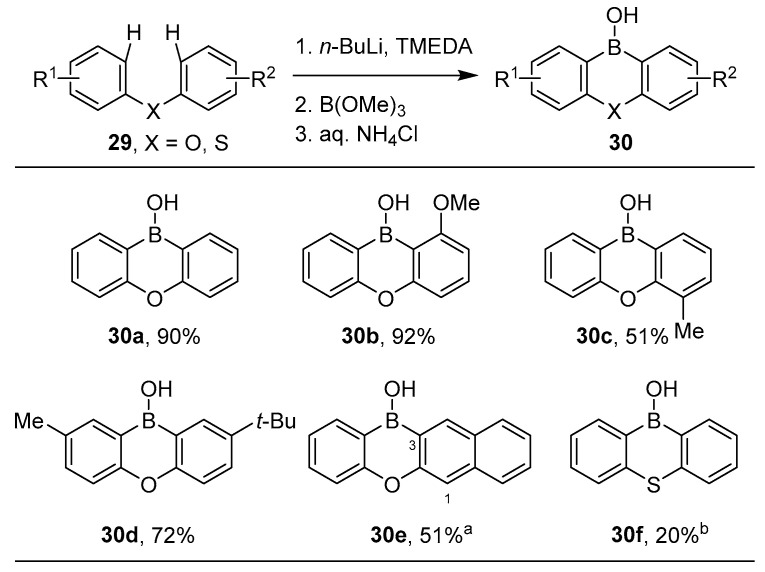
Synthesis of cyclic borinic acids by *ortho*-metalation. (a) Traces of the C1 regioisomer were observed. (b) B(O*n*-Bu)_3_ was used.

An alternative synthesis of dibenzoxaborininols was developed by Fu et al. who proposed a double *ortho*-metalation of diarylethers **29** (or thioethers) with *n*-butyllithium/TMEDA, followed by trapping with trimethyl borate (Scheme 9) [39]. The method has been exemplified with more than twenty substrates, including symmetrical **30a** and non-symmetrical borinic acids (**30b–e**) bearing alkyl (i.e., Me, Et, *t*-Bu) and methoxy substituents. It is worth mentioning that in the case of **30b**, the regioselectivity is dictated by the lithiation step, which occurs between the MeO and ArO group thanks to an additional coordination of the organolithium reagent. In the naphthalene series (i.e., **30e**), however, a competitive deprotonation at the C1 and C3 positions takes place, with the latter always observed as the major regioisomer. It is interesting to note that these cyclic borinic acids were successfully transformed into dibenzofuran derivatives under palladium catalysis. The same strategy was applied to diaryl thioether, although the corresponding thia-boraanthracene **30f** was isolated with only a 20% yield, reflecting the lower ability of sulfur to direct *ortho*-metalation [36].

**Scheme 10:**
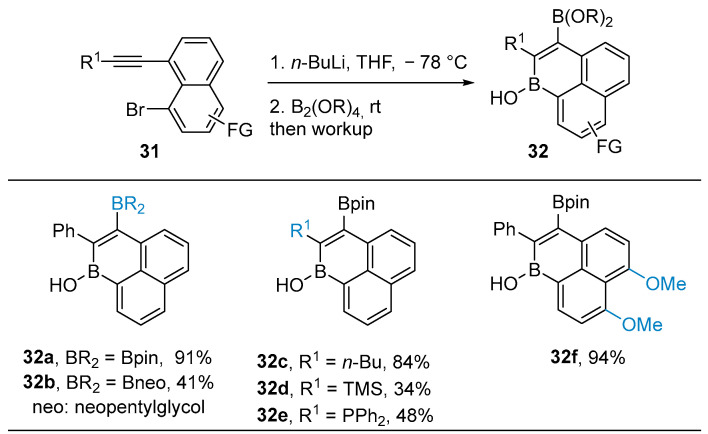
Synthesis of boron-doped PAHs **32** by nucleophilic diborylation of alkynes **31**.

In 2020, Uchiyama et al. described an original nucleophilic diboration of alkynes to build up boron-doped PAHs **32** (Scheme 10) [40]. Bromo-naphthalenes **31** were converted into the aryl lithium species with *n*-BuLi at low temperature, then reacted with bis(pinacolato)diboron at room temperature. After workup, cyclic borinic acids **32** were obtained in modest to excellent yields, depending on the alkyne, the aromatic substitution and the nature of the diboron reagent.

**Scheme 11:**
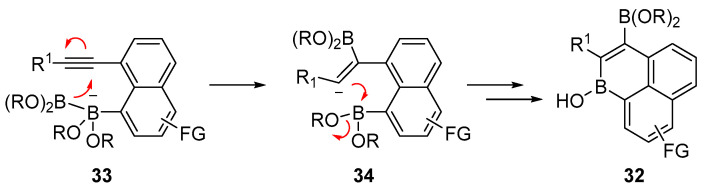
Proposed mechanism for the diborylation of alkynes.

The reaction proceeds by formation of a sp^2^-sp^3^ diboron intermediate **33**, which undergoes intramolecular nucleophilic attack of the formal boryl anion onto the suitably positioned triple bond (Scheme 11). Consecutive intramolecular addition of the newly formed carbanion (i.e., **34**) on the boronic ester led to the cyclized product **32** after hydrolysis.

#### 2.1.2. Use of Borontrihalides

**Scheme 12:**
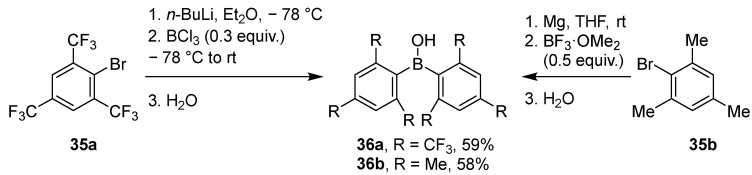
Synthesis of diarylborinic acids using BCl_3_ and BF_3_·OMe_2_.

In comparison to trialkoxyboranes, the corresponding boron trihalide reagents are significantly more reactive due to the enhanced Lewis acidity at the boron centre. These reagents are thus particularly adapted for the synthesis of diarylborinic acids bearing hindered *ortho*-substituents or electron-withdrawing groups. As highlighted in Scheme 12, borinic acid **36a** substituted by electron-deficient CF_3_ groups was prepared from **35a** by addition of the corresponding organolithium species onto BCl_3_, followed by hydrolysis [41]. A detailed study of this reaction showed that a chlorine/fluorine exchange (from the CF_3_ substituent) occurred during the reaction to afford a mixture of Ar_2_BCl and Ar_2_BF [42]. Hydrolysis afforded borinic acid **36a** in a 59% isolated yield even though both fluoro- or chloro-diarylboranes were formed. The analogous dimesityl borinic acid **36b** was obtained with a 58% yield by the addition of the Grignard reagent of **35b** on boron trifluoride dimethyl etherate [43].

**Scheme 13:**
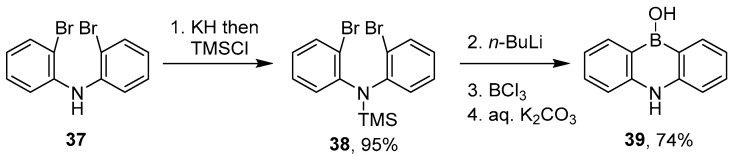
Synthesis of azaborine **39** using BCl_3_ as borylating agent.

Access to the azaborine framework **39**, bearing a free amine, has been described by using BCl_3_ as the borylating agent [44]. The authors showed that direct conversion of **37** in azaborine **39**, by treatment with *n*-BuLi, then BCl_3_, resulted in a low yield (21%) (not shown). A more efficient stepwise method was then developed, consisting of prior protection of the aniline **37** by a TMS group followed by a lithiation/borylation/hydrolysis sequence. Overall, the isolated yield of azaborine **39** was significantly increased to 70% (Scheme 13).

**Scheme 14:**
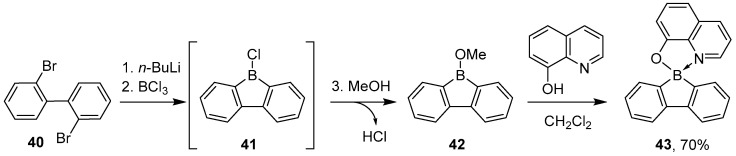
Synthesis of borafluorene derivative **43**.

Spiro-borafluorene complexes (i.e., **43**) have been studied for their particular structural features, perpendicular alignment between the ligand and the boracycle, which influence their physicochemical properties. As depicted in Scheme 14, the borafluorene unit can be prepared from biphenyl bromide **40**, by a double lithiation followed by subsequent trapping with BCl_3_. Methanolysis of chloroborane **41** gave **42** and HCl as a byproduct, which was removed under vacuum. Treatment of **42** with 8-hydroxyquinoline then gave compound **43** with a 70% yield [45]. It is worth mentioning that direct reaction of the intermediate dilithium species with B(OMe)_3_ affords the diborylated compound (not shown) instead of the boracycle.

**Scheme 15:**
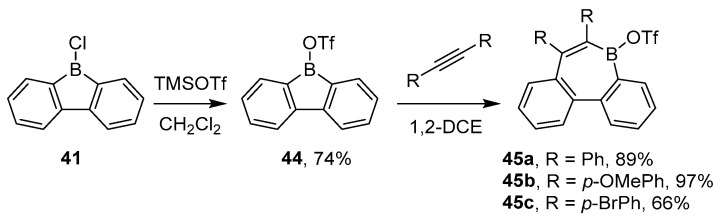
Synthesis of borepinol derivatives by carboborylation of alkynes.

Fukushima et al. showed that chloroborafluorene **41** can be transformed into the trifluoromethane sulfonyloxy analog **44** by treatment with TMSOTf (74% yield) (Scheme 15) [46]. This highly reactive intermediate was then engaged in a 1,2-carboboration of alkynes to produce borepines **45a–c** in excellent yields. DFT calculations indicate that borafluorene and diphenylacetylene first form a complex by coordination of the alkyne to the lone orbital of the boron. This complex then evolves into the formation of the borepine **45**, by a four-membered transition state, resulting in the alkyne insertion in the B-C bond.

**Scheme 16:**
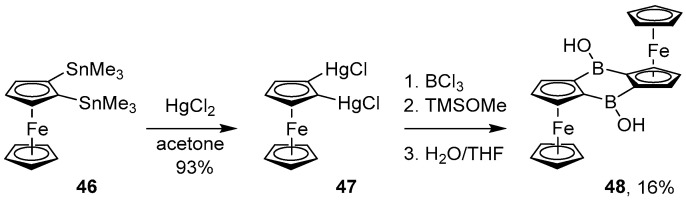
Synthesis of diboradiferrocene **48**.

During the course of their study on novel bifunctional organoboranes, Jäkle et al. designed diboradiferrocene **48** bearing a bis-borinic acid backbone (Scheme 16). In their initial studies, they observed that direct transmetalation of 1,2-bis(stannyl)ferrocene **46** with boron halides resulted in a rearrangement that produced 1,1′-bis(diboryl)ferrocene (not shown). This issue was circumvented by first reacting **46** with HgCl_2_ (i.e., **47**), then with BCl_3_ [47]. The dichloroborane intermediate was next subjected to methanolysis with TMSOMe, to avoid the formation of HCl and prevent decomposition. Hydrolysis eventually led to bis(borinic acid) **48** with a 16% yield [48].

#### 2.1.3. Use of Aminoboranes

**Scheme 17:**
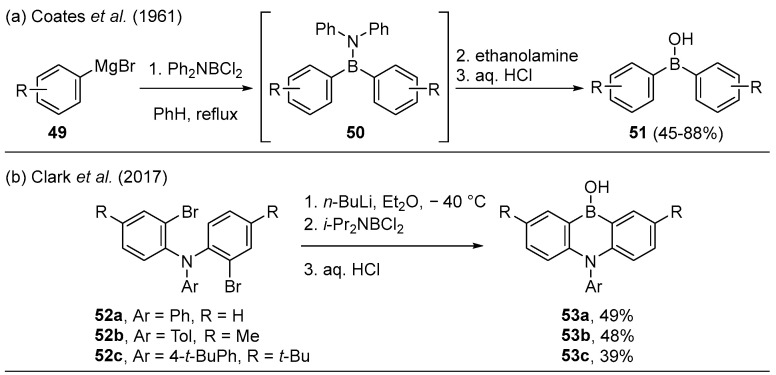
Synthesis of borinic acids **51** and **53a–c** using aminodichloroboranes. (**a**) [49]; (**b**) [22].

Despite many applications of B(OR)_3_ and BX_3_ as borylating agents, their main drawback relates to the difficulty of controlling the number of aryl residues transferred to the boron centre (see Scheme 2). In the early sixties, Coates et al. described the use of diarylaminodichloroboranes (Cl_2_B-NAr_2_) for the selective preparation of diarylborinic acids (Scheme 17a) [49]. These borylating agents display two important features: (1) they are monomeric in solution when the aryl is a sterically hindered group (Ar = Ph, *o*-Tol or Mes), and (2) the presence of the amino group on the boron reduces its Lewis acidity by donation of the nitrogen lone pair to the boron vacancy. These properties give a borylation agent in which the boron atom is electrophilic enough to react with two equivalents of Grignard reagents, but prevent the third addition. Thus, with only a slight excess of arylmagnesium bromide **49**, diarylborinic acids **51** were obtained in yields ranging from 45% to 88%. In 2017, Clark et al. illustrated the synthesis of cyclic borinic acids **52a–c** by a similar strategy. While reaction of the dilithium intermediate with B(OR)_3_ reagents resulted in low yields, the authors found that the use of dichloro(diisopropylamino)borane led to improved yields of azaborines **53a–c** (Scheme 17b) [22].

**Scheme 18:**
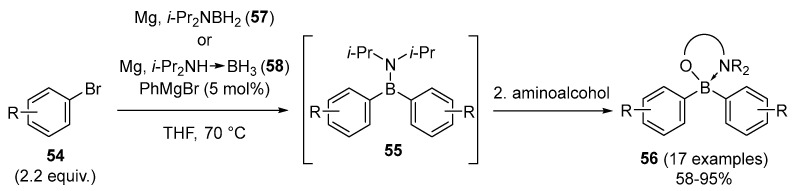
Synthesis of symmetrical borinic acid derivatives **56**.

Our group proposed diisopropylaminoborane **57** (DIPOB) as the borylating agent for the synthesis of symmetric and unsymmetrical borinic acid derivatives under non-cryogenic conditions. The reaction selectively gives diarylborinic acids even when a slight excess of arylbromide **54** (2.2 equivalents) is employed (Scheme 18) [50,51]. No boron impurities were observed, and the products were obtained by precipitation without the need of flash chromatography. Although DIPOB is easy to synthesize from low cost starting materials, this air and water sensitive reagent needs to be prepared by the thermolysis of diisopropylamine–borane complex **58** (DIPAB) and stored under an inert atmosphere. As a convenient alternative, we showed that DIPOB can be easily in situ generated from water- and air-stable DIPAB, by treatment with a catalytic amount of a Grignard reagent [52]. Overall, both methods gave borinates **56** in good to excellent yields after precipitation of the borinic acids with a suitable amino alcohol.

**Figure 2:**
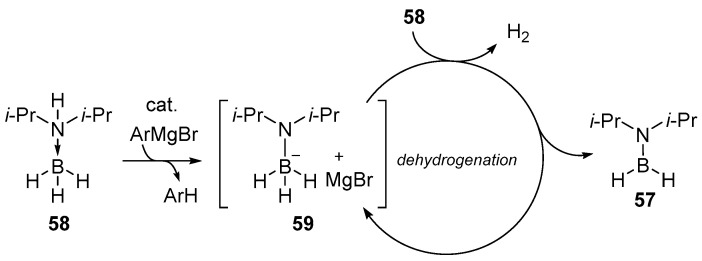
Proposed mechanism for dehydrogenation of DIPAB **58**.

Regarding the dehydrogenation of DIPAB, we hypothesized that, in the initial step, the Grignard reagent deprotonates DIPAB **58** to give aminoborohydride **59**. The latter is basic enough to undergo deprotonation of DIPAB **58**, generating DIPOB **57**, aminoborohydride **59** and hydrogen as the sole side product (Figure 2).

### 2.2. By Organometallic Catalysis with Boron Reagents

**Scheme 19:**
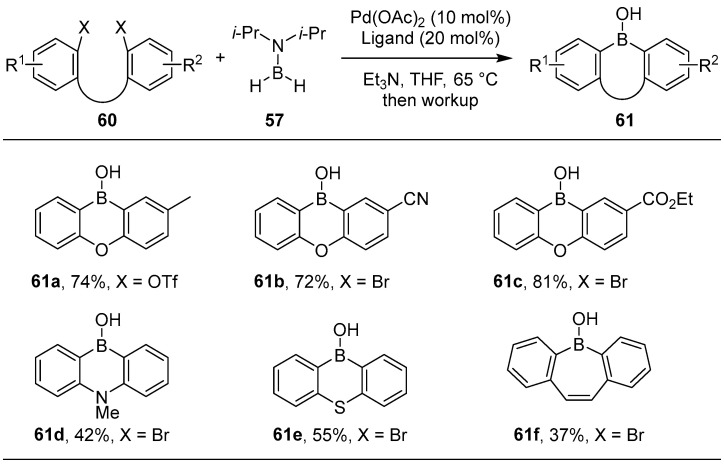
Synthesis of cyclic borinic acids catalyzed by palladium with DIPOB **57**.

The syntheses of diarylborinic acids described so far rely on organolithium or organomagnesium reagents that considerably narrow down the functional groups’ compatibility. Chatani et al. recently reported the palladium-catalyzed synthesis of cyclic borinic acids from diarylbromides or triflates **60** and DIPOB **57** [33]. The main advantage of this catalytic strategy is undoubtedly the large tolerance towards sensitive functional groups providing access to symmetrical and non-symmetrical cyclic borinic acids bearing ester, nitrile, etc. (Scheme 19). Furthermore, borinic acids that embedded oxygen (**61a–c**), nitrogen (**61d**), sulfur (**61e**) or alkenyl (**61f**) tethers were prepared in good yields when compared to the known methods.

**Figure 3:**
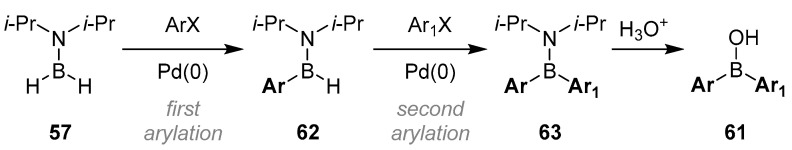
Proposed mechanism of the Pd-catalyzed diarylation of aminoboranes.

The reaction proceeds through a sequential diborylation process (Figure 3), similarly to organolithium reagents. It is known that the reaction of arylhalides with DIPOB under palladium catalysis leads exclusively to arylaminoborane **62**, due to steric congestion of the diisopropyl amine [53]. However, in this particular case, an intramolecular reaction probably facilitates the second borylation, leading to **63**, and then **61** after hydrolysis.

**Scheme 20:**
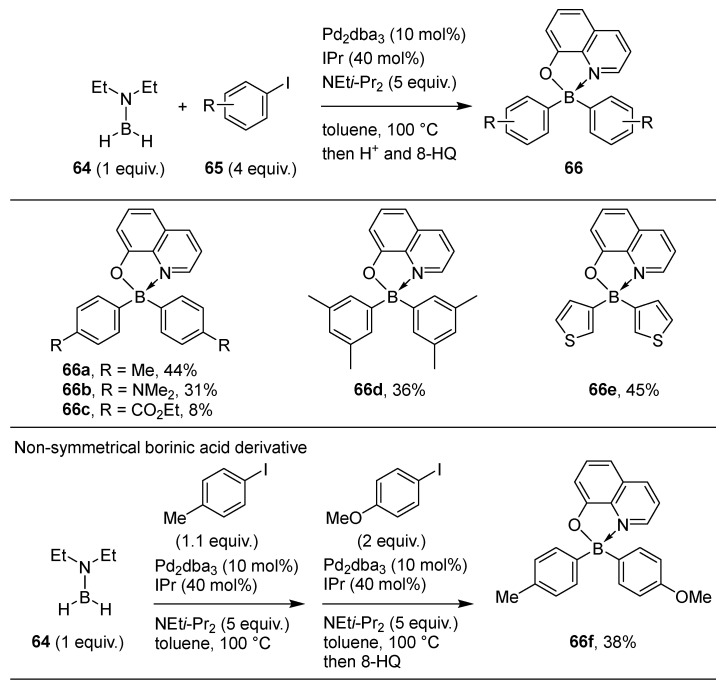
Borinic acid synthesis by palladium-catalyzed diarylation of diethylaminoborane.

Later on, the approach was extended to the synthesis of acyclic borinic acid derivatives by the same group [54]. As mentioned above, the use of DIPOB only resulted in the formation of boronic acids under palladium catalysis. Thus, after the screening of various substituted aminoboranes, and investigating their aggregation state (monomer versus dimer form), they found that the less bulky diethylaminoborane reagent **64** affords a good compromise in terms of reactivity at 100 °C (Scheme 20). Under optimized conditions, symmetrical and non-symmetrical borinic quinolates **66** were obtained in modest yields. Interestingly, the reaction is compatible with an ester moiety (i.e., **66c**), although the isolated yield is pretty low.

### 2.3. By Electrophilic Aromatic Substitution with Boron Reagents

Borylation by electrophilic aromatic substitution is an alternative route to prepare organoboron compounds, which has been applied in few examples for the synthesis of borinic acids. The regioselectivity of the SE_Ar_ is often controlled by a combination of steric and electronic properties of the substituents on the aromatic moiety. When arylstannanes or arylsilanes substrates are used, the boron electrophile reacts at the ipso position, resulting in an exchange of the Sn/Si by a boron atom.

**Scheme 21:**
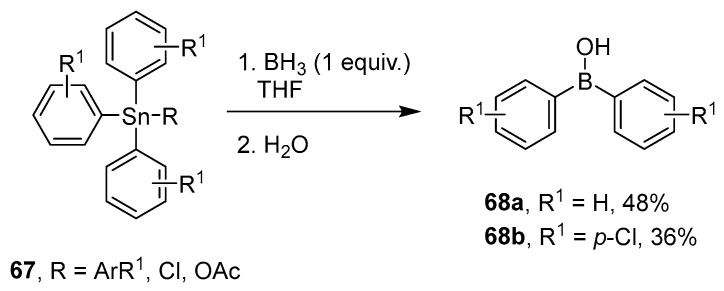
Synthesis of borinic acids by tin–boron transmetalation.

In an early report in 1984, Thorpe et al. showed that diboranes react with arylstannanes **67**, to provide symmetrical borinic acids **68**, in modest yields, after hydrolysis (Scheme 21) [55]. Usually, a mixture of boronic and borinic acid was observed, depending on the substitution pattern and the BH_3_/substrate ratio.

**Scheme 22:**
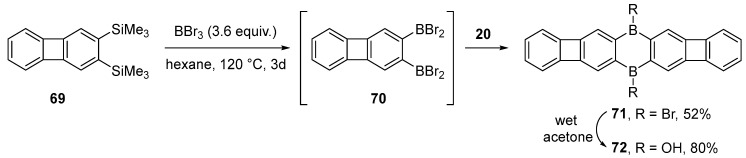
Synthesis of bis-borinic acid **72** by Si/B transmetalation.

A two-step intermolecular process was recently described by Wagner et al. for the synthesis of polycyclic bis-borinic acid **72**, a compound developed for its optoelectronic properties. The first transmetalation takes place by reacting bis-silane **69** with BBr_3_ at 120 °C, to produce bis-dibromoborane **70** (Scheme 22) [56,57]. The latter undergoes a second transmetalation reaction with **69**, leading to the desired bis-bromoborane **71** with a 52% yield. The corresponding borinic acid **72** was then obtained with an 80% yield by hydrolysis in wet acetone.

**Scheme 23:**
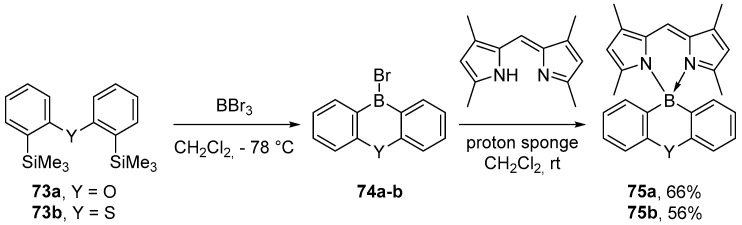
Synthesis of **75a–b** by Si/B transmetalation.

A similar strategy was used by the group of Wang to prepare spiro-BODIPYs **75a–b**, starting from tethered diarylsilanes **73a–b** (Scheme 23) [58,59]. Treatment of **73a–b** with BBr_3_ afforded the cyclic diarylbromoboranes **74a–b** by two consecutive inter-, then intramolecular transmetalation steps. Subsequent reaction with in situ generated dipyrromethene led to the spiranic compounds **75a–b** in good yields. Of course, the corresponding borinic acids could also be synthesized by the hydrolysis of compounds **74a–b** as exemplified in Scheme 13.

**Scheme 24:**
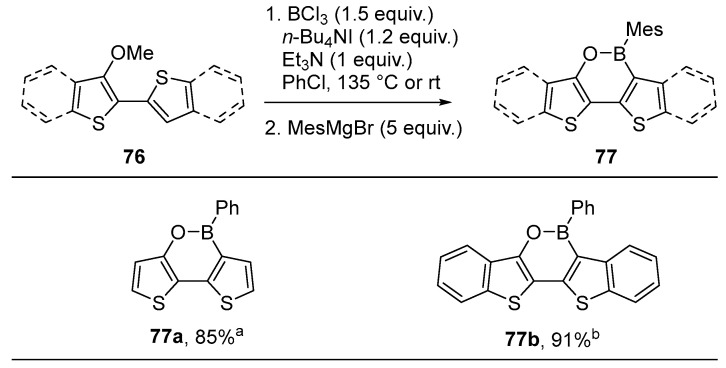
Synthesis of mesityl-substituted borinic ester **77a–b** by electrophilic borylation. (a) Reaction performed at 135 °C. (b) Reaction performed at room temperature.

Despite transmetalation of arylsilanes and stannanes, the directed electrophilic C–H borylation is an effective way of controlling the regioselective functionalization of arenes, heteroarenes or alkenes under metal-free conditions. In this regard, Suga et al. reported the C−H borylation of bis-thiophene derivatives **76** using BCl_3_ (Scheme 24) [60]. The authors showed that the tandem demethylation and subsequent Friedel–Craft-type borylation was effective in the presence of BCl_3_, triethylamine, and *n*-BuN_4_I. Thus, by carrying out the reaction at 135 °C (i.e., **77a**) or rt (i.e., **77b**) in PhCl, the intermediate chloroborane is formed, and directly treated with mesityl Grignard reagent to give **77a–b** with excellent yields.

**Scheme 25:**
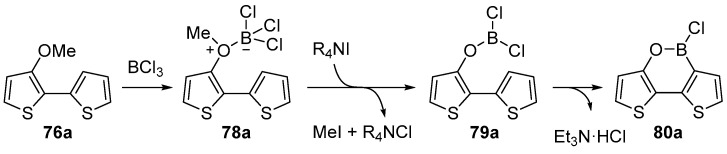
Proposed mechanism of electrophilic borylation of **76a**.

The proposed mechanism starts by complexation of the boron atom to the methoxy group to produce **78a** (Scheme 25). The demethylation process is then favored by the iodide ion, affording dichloroborane **79a**. Subsequent intramolecular borylation gave **80a**, which was reacted with MesMgBr to obtain **77**.

**Scheme 26:**
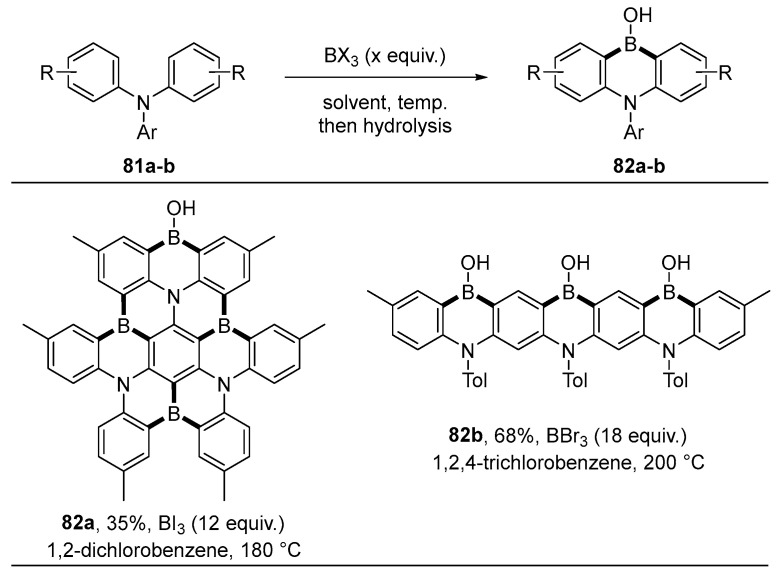
Electrophilic borylation of triarylamines **81a**–**b**.

Electrophilic borylative cyclization of triarylamines is a straightforward strategy to prepare BN-doped polycyclic aromatic hydrocarbons (Scheme 26). Hatakeyama et al. showed that the reaction of 12 equivalents of BI_3_ with polysubstituted 1,3,5-triarylamine **81a** resulted in a quadruple borylation process [61]. The target borinic acid **82a** was isolated with a 35% yield, albeit with 3% of triborylated derivative and a large amount of side products. Other boron trihalide reagents (BCl_3_ or BBr_3_) did not react in this particular example. Colman and his group recently described the synthesis of triborinic acid **82b** in the presence of 18 equivalents of BBr_3_ at 200 °C [62]. Even though these conditions are pretty harsh, triborinic acid **82b** was isolated with a good 68% yield.

**Scheme 27:**
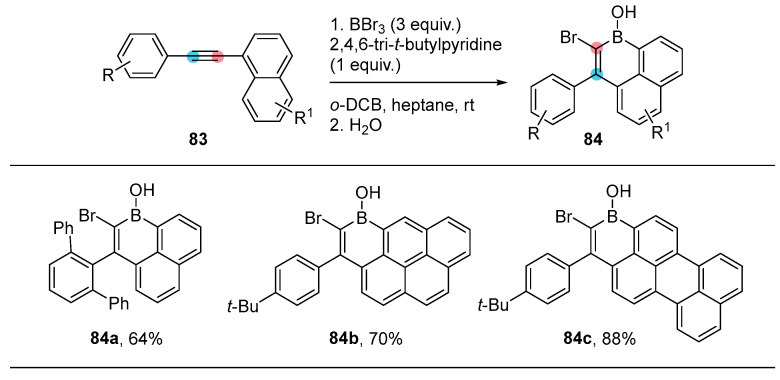
Synthesis of extended boron-doped PAH **84** by electrophilic borylation.

Ingleson and coworkers developed a synthesis of 1-boraphenalenes relying on the electrophilic borylation of alkynes **83**, substituted with appropriate aromatic rings (Scheme 27) [63,64]. Initially, borylation with BBr_3_ resulted in the formation of the rearranged product **84** after hydrolysis, along with byproducts derived from the addition of HBr to the triple bond (HBr is formed after the S_E_Ar reaction). By adding 2,4,6-tri-t-butylpyridine to the reaction, extended boron-doped polycyclic aromatic hydrocarbons (PAHs) **84** were synthesized in good yields.

**Scheme 28:**
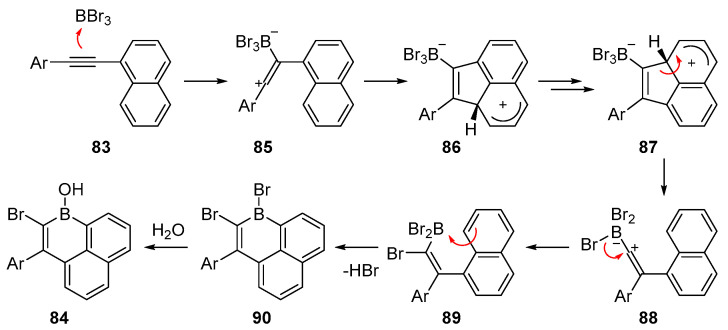
Proposed mechanism for the borylative cyclisation of **83**.

The unexpected formation of polycyclic borinic acids **84** was rationalized by evoking first an electrophilic borylation of the alkyne **83** to form **85** (Scheme 28). The subsequent intramolecular Friedel–Craft reaction onto vinylic carbocation (**85** to **86**), tautomerization (**86** to **87**), and then C–C bond cleavage results in carbocation **88** that undergoes 1,2-migration of bromide (i.e., **89**). Intramolecular electrophilic borylation (i.e., **90**) and hydrolysis provide cyclic borinic acid **84**.

**Scheme 29:**
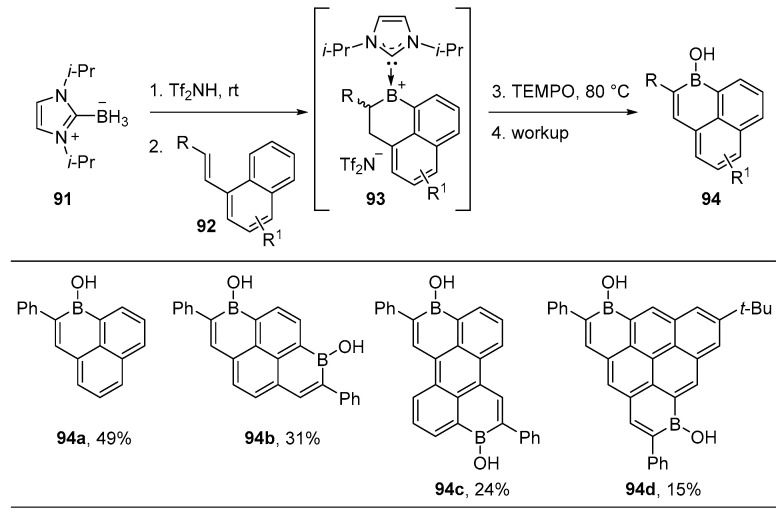
Synthesis of cyclic borinic acids **94** via electrophilic borylation with NHC-borenium.

In addition to the use of trihalogenoboranes in electrophilic C–H arene borylation, it has recently been reported that NHC-borenium is also effective in this transformation [65,66]. Three-coordinate NHC-borenium cations are strongly electrophilic boron compounds that can be generated from the air- and moisture-stable NHC-boranes and a strong acid. Würthner et al. recently developed a synthesis of cyclic borinic acids relying on this chemistry study (Scheme 29); the NHC-borenium species is first generated from 1,3-diisopropylimidazol-2-ylidene borane **91** and HNTf_2_ [67,68,69,70]. The subsequent addition of **92**, led to the cyclic NHC-borane intermediate **93**. The reaction pathway follows a sequential regioselective hydroboration of alkenes and C–H borylation of the aromatic scaffold. After dehydrogenation of **93** under oxidative conditions and aqueous workup, borinic and bis-borinic acids **94a–d** were isolated in modest yields ranging from 15% to 49%.

**Scheme 30:**
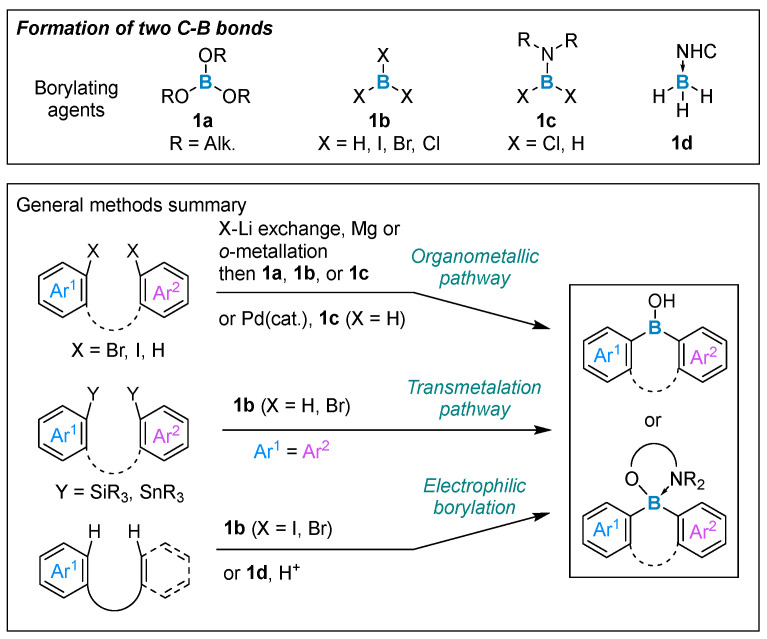
Overview of the synthetic methods relying on the formation of two C-B bonds.

To summarize, an overview of the different borylating agents and general methods to synthesize borinic acid derivatives through the formation of two C-B bonds is given in Scheme 30. Overall, these approaches have been used to prepare a wide range of acyclic, cyclic, symmetric and non-symmetric borinic acids, and their corresponding chelates. The field of applications of these compounds mainly lies in catalysis, cross-coupling, medicinal chemistry, sensors, polymers and luminescent devices.

## 3. Formation of One Carbon–Boron Bond

### 3.1. By Nucleophilic Addition of ArLi and ArMgX to Aryl Boron Reagents

The addition of organolithium or Grignard reagents to arylboranes is closely related to the previous methods using trialkylborates or trihalogenoboranes. The main advantage of this approach is the possibility to introduce two different aryl substituents at the boron, allowing for the synthesis of non-symmetrical borinic acids.

**Scheme 31:**
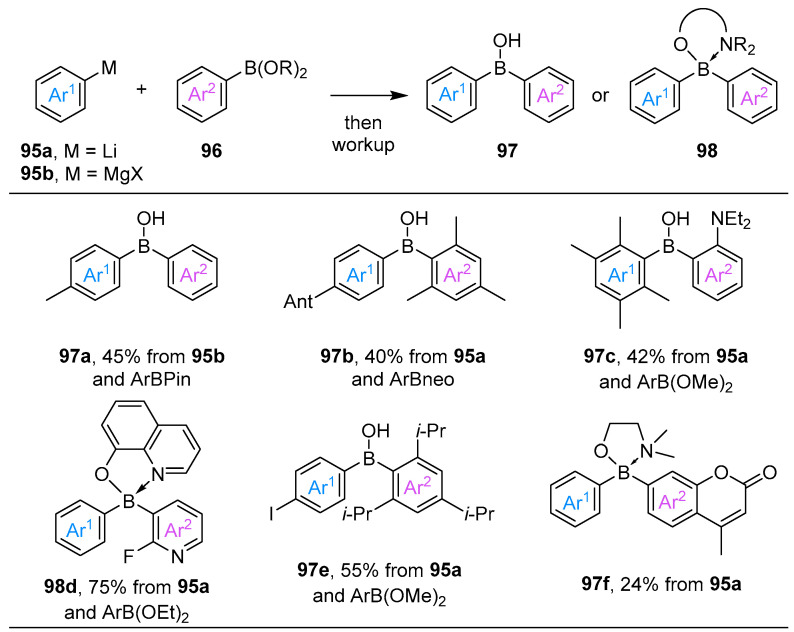
Synthesis of acyclic non-symmetrical borinic acids by organometallic addition on boronic esters.

As shown in the examples in Scheme 31, the nucleophile can be either aryllithium **95a** or arylmagnesium halide **95b**, which reacts with boronic esters **96** bearing pinacol, neopentyl glycol or alcohol (OMe, OEt) ligands.

For example, the reaction tolerates aromatic rings substituted with methyl, aryl or amino groups (**97a–c**) on the organometallic reagent **95** or the boronic acid partner **96** [17,71,72]. The addition of aryllithium onto heteroaromatic boronic esters also performs well, as shown with pyridine-substituted boron quinolate **98d** obtained with a good yield [73,74]. Even more interesting is the possibility of incorporating an iodine atom such as in borinic acid **97e** [75]. In brief, monolithiation of 1,4-diiodobenzene, followed by the addition onto the corresponding boronic ester, led to **97e** that can be further functionalized by Still coupling thanks to the remaining iodine substituent. This method was also used to prepare borinic acid **97f** derived from coumarin, a new probe to monitor H_2_O_2_-mediated signaling processes [76].

**Scheme 32:**
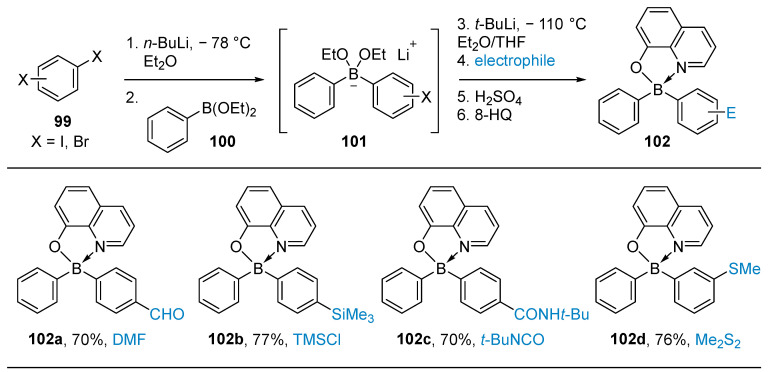
Synthesis of non-symmetrical borinic acid derivatives **102** through lithium “ate” complexes.

Dihalogenobenzenes have also been used in the one-pot synthesis of functionalized non-symmetrical borinic acid derivatives (Scheme 32) [77]. In this approach, diiodo- or dibromo arenes **99** are treated with one equivalent of *n*-BuLi, then the corresponding aryllithium is added to the phenyl boronic ester **100** at −78 °C. A second halogen–lithium exchange takes place by reacting the resultant “ate” complex **101** with *t*-BuLi at −110 °C. The dianionic species (not shown) is then trapped with various electrophiles (DMF, isocyanate, chlorosilane, disulfide) to provide unsymmetrical boron quinolate complexes **102** with good isolated yields.

**Scheme 33:**
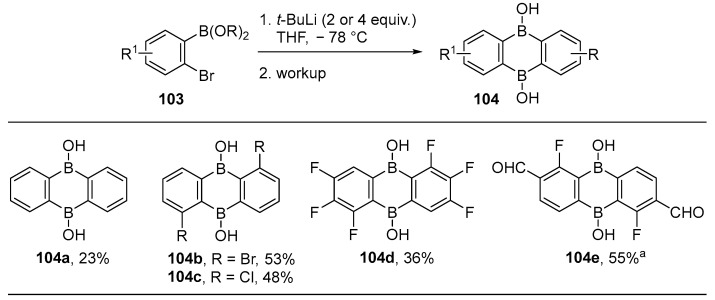
Synthesis of 9,10-diboraanthracenes **104**. (a) From boronic ester **103e**, R^1^ = CH(OMe)_2_.

The synthesis of 9,10-diboraanthracenes **104** has been described by the same group, but this time starting from 2-bromophenyl boronate esters **103** (R = Me, Et, *i*-Pr) (Scheme 33) [78]. For instance, the reaction of **103** performed with an excess of *t*-BuLi (2 or 4 equivalents) at low temperature provided borinic acids **104** in modest yields [79].

**Scheme 34:**
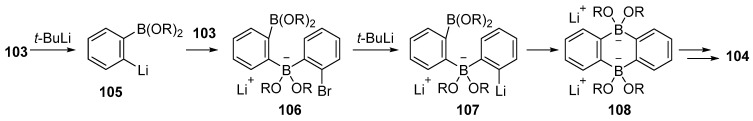
Proposed mechanism for the formation of 9,10-diboraanthracenes **104**.

Scheme 34 provides a mechanistic proposal for the formation of **104**. In line with the computational studies they performed, the authors showed that the Br/Li exchange with *t*-BuLi to produce **105** is kinetically favored over nucleophilic attack at the boron atom. The lithium adduct **105** then reacts with the remaining starting boronic ester **103** to form “ate” complex **106**. A second Br/Li exchange (i.e., **107**) followed by subsequent cyclization (i.e., **108**) afforded diborinic acids **104** after hydrolysis. Although an alternative pathway involving the dimerization of **105** was proposed, it seems unlikely due to the low concentration of the lithiated boronate species **105** under the developed protocol.

**Scheme 35:**
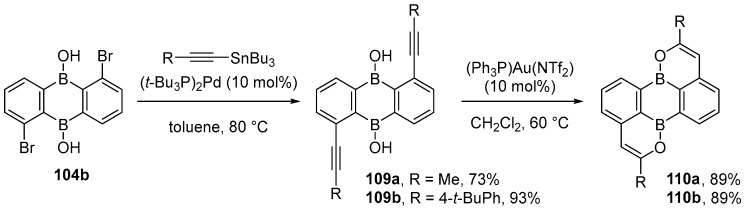
Synthesis of polycyclic borinic esters **110** by gold-catalyzed *h*ydroalkoxylation of alkynes.

An interesting synthetic application of these compounds was highlighted by Wagner and coworkers who designed bis-BO-perylene derivatives from 9,10-diboraanthracenes **104b** (Scheme 35) [80,81,82]. In short, bis-alkynylation of **104b** by a Stille-type coupling (i.e., **109**) followed by gold-catalyzed twofold cyclisation of the borinic acid **109** onto the triple bond, produced boron-based perylenes **110a–b** in excellent 89% yields. These compounds undergo reversible reduction, and could be used for organic electronics.

**Scheme 36:**
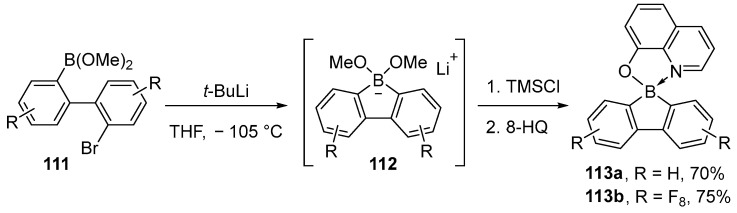
Synthesis of borinic acid derivatives **113a–b** by intramolecular borylation.

An intramolecular variation was proposed recently to prepare strained spiro boron quinolates **113** (Scheme 36) [45]. Under the same conditions as Scheme 33, bromo boronic ester **111** was converted into the lithium “ate” complex **112**. After quenching with TMSCl, borinic acids were isolated in good yields as the quinolate complexes **113a–b**.

**Scheme 37:**
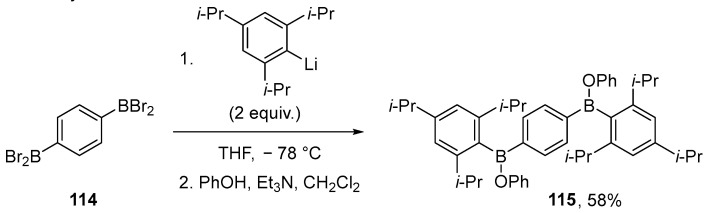
Synthesis of diborinic ester **115**.

Aryl halogenoboranes can also be used as precursors of non-symmetrical borinic acid derivatives, as exemplified by Bolm and Helten during the course of their study on BO-containing hybrid polymers [83,84]. A model substrate, *para*-substituted bis-dibromoborane **114**, was reacted with lithium 2,4,6-triisopropylphenyl (TipLi) to give the corresponding bis-diarylbromoborane (Scheme 37). The authors then tried to form the B-O bond in the presence of silylated phenol PhOSiMe_3_. However, the Si/B exchange was very slow and the conversion was not complete even after several weeks. The reaction with PhOH and trimethylamine appeared to be much cleaner, leading to **115** with a 58% overall yield.

**Scheme 38:**
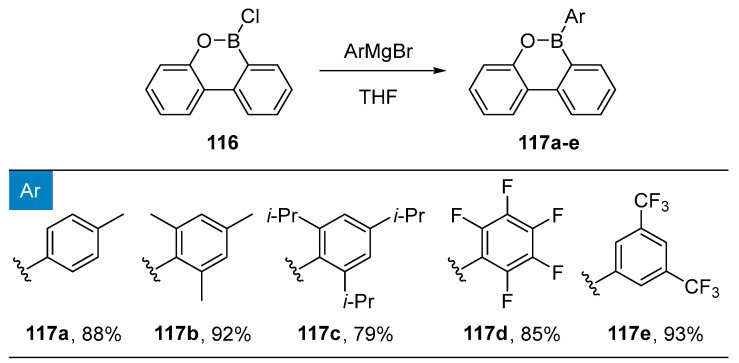
Synthesis of 10,9-oxaboraphenanthrenes **117**.

The addition of organolithium and Grignard reagents on 9-chloro-oxaboraphenanthrene **116** was investigated as a route to borinic ester derivatives (Scheme 38). Both nucleophiles were convenient in this transformation; however, aryllithium led to a mixture of single (i.e., **117**) and double additions to form the lithium borate (not shown). In contrast, Grignard reagents gave compounds **117a–e** with excellent isolated yields, even for those bearing sterically hindered group (**117c**) or electron-deficient substituents (**117d–e**) [85].

**Scheme 39:**
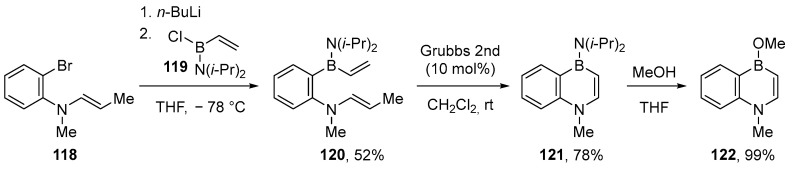
Synthesis of methoxy-substituted 1,4-azaborazine **122**.

An original synthesis of 1,4-azaborazine has been described starting from enamine **118** (Scheme 39), obtained in three steps from 2-bromo aniline [86]. Bromide–lithium exchange followed by trapping with vinylchloroaminoborane **119** gave diene **120**. Ring closure metathesis with Grubbs’ 2nd generation catalyst and methanolysis quantitatively delivered borinic ester **122**. This compound was used as a platform for the design of ligands incorporating the 1,4-azaborine motif.

### 3.2. By Electrophilic Aromatic Substitution with Aryl Boron Reagents

**Scheme 40:**
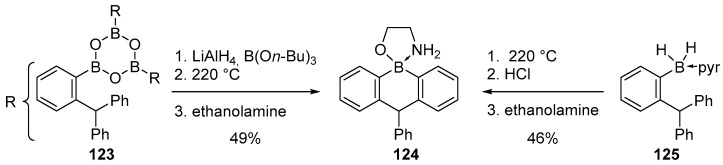
Synthesis of **124** by pyrolysis of organoboranes **123** and **125**.

Early examples of borinic acid synthesis via intramolecular electrophilic borylation were reported by Bickelhaupt and coworkers in 1972 (Scheme 40). The reaction was carried out by pyrolysis at 220 °C of boroxine **123**, in combination with lithium aluminum hydride and tributoxyborane, to afford borinate **124** with a 49% yield after treatment with ethanolamine [87]. The authors proposed that the reaction may occur through the formation of a primary aryl borane (ArBH_2_), or a butoxy derivative such as ArB(H)(O*n*-Bu) or ArB(O*n*-Bu)_2_. Alternatively, the same product was obtained with a similar yield by pyrolysis of the pyridine complex **125** [88].

**Scheme 41:**
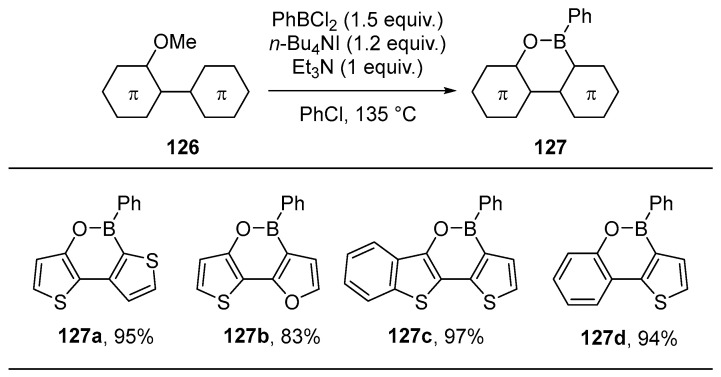
Synthesis of BO heterocycles **127** by electrophilic borylation with PhBCl_2_.

As described before, recent developments in the field of electrophilic borylation rely on the use of more reactive boron species (BX_3_, ArBX_2_, etc.) to perform the reactions in practical conditions of temperature. In this regard, Suga et al. reported the C−H borylation of thiophene derivatives using PhBCl_2_ (Scheme 41) [60]. Although the reaction works well on the corresponding hydroxyl analog of **126**, the starting material was difficult to obtain and unstable. By contrast methoxythiophene derivatives **126** are easy to prepare and handle, and their synthesis is versatile. The authors showed that the treatment of thiophene **126** with PhBCl_2_ alone did not result in the cyclized product. However, the tandem demethylation and subsequent Friedel–Craft-type borylation was effective in the presence of triethylamine and a stoichiometric (or catalytic) amount of *n*-BuN_4_I. Thus, by carrying the reaction at 135 °C in PhCl, a series of dithieno-1,2-boxaborines **127a–d** was obtained in excellent yields whatever the nature of the second aromatic ring. The mechanism is related to the one already described in Scheme 25.

**Scheme 42:**
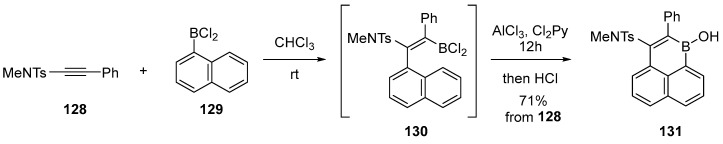
Synthesis of borinic acid **131** by sequential arylboration/electrophilic borylation.

In the course of their study on 1,2-carboboration of ynamides with aryl dichloroboranes, Studer et al. described the one-pot synthesis of boraphenalene **131** (Scheme 42) [89]. Regioselective borylation of ynamide **128** with napththyl dichloroborane **129** in chloroform furnished intermediate **130**. Subsequent treatment with AlCl_3_ and dichloropyridine provided boraphenalene **131** with a 71% yield by intramolecular boron Friedel–Crafts arylation.

**Scheme 43:**
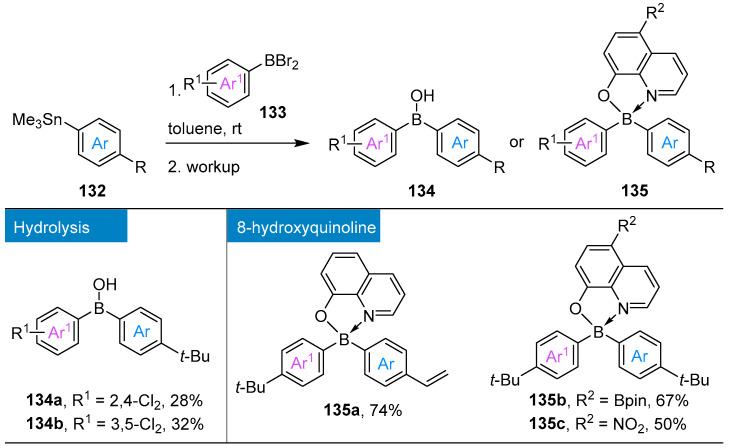
Synthesis of borinic acid derivatives **134–135** by transmetalation with ArBBr_2_.

As seen in the previous section, the transmetalation of arylstannanes is a powerful way of controlling the regioselectivity of the borylation of BX_3_. Within the framework of borinic acid synthesis, the method was extended to the reaction with ArBBr_2_ (Scheme 43) [90,91,92]. Several electron-deficient borinic acids **134a–b** or boron quinolate complexes **135a–c** were obtained in modest to good yields. The reaction seems compatible with a range of functional group such as nitro, boronic ester, vinyl, etc.

### 3.3. Other Methods

**Scheme 44:**
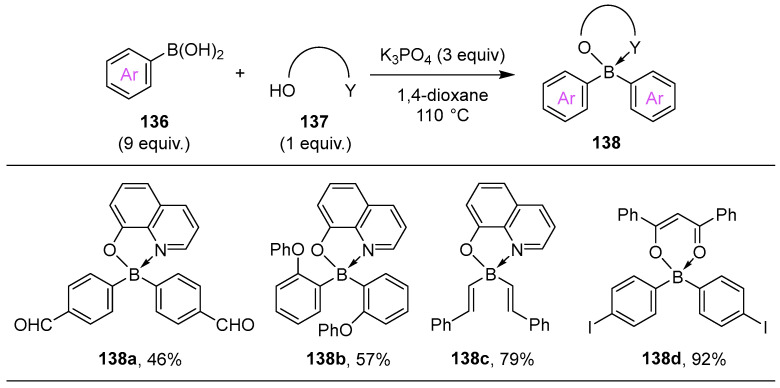
Ligand-promoted synthesis of boron(III) complexes **138**.

As seen previously, one of the main methods to prepare borinic acid derivatives relies on the use of organometallic reagents (ArLi or ArMgX), which strongly limits the functional group tolerance. Recently, Lee and coworkers reported a metal-free one-pot synthesis of tetracoordinated borinic acids (Scheme 44) [93]. The optimal conditions require nine equivalents of boronic acids **136**, in the presence of a bidentate ligand **137** and K_3_PO_4_ (3 equiv.) in refluxing 1,4-dioxane. The reaction performs well with substrates incorporating electron-withdrawing (**138a**) or -donating (**138b**) groups on the aromatic ring. Remarkably, styryl boronic acid led to the corresponding boron quinolate **138c** with a good 79% yield. The scope of the reaction was also extended to several *N,O*, *N,N* and *O,O* bidentate ligands as exemplified with compound **138d** bearing a β-diketonate ligand. Preliminary mechanistic studies suggest that the reaction proceeds thought a boronic anhydride intermediate, with the formation of an “ate” complex and concomitant organic group transfer.

**Scheme 45:**
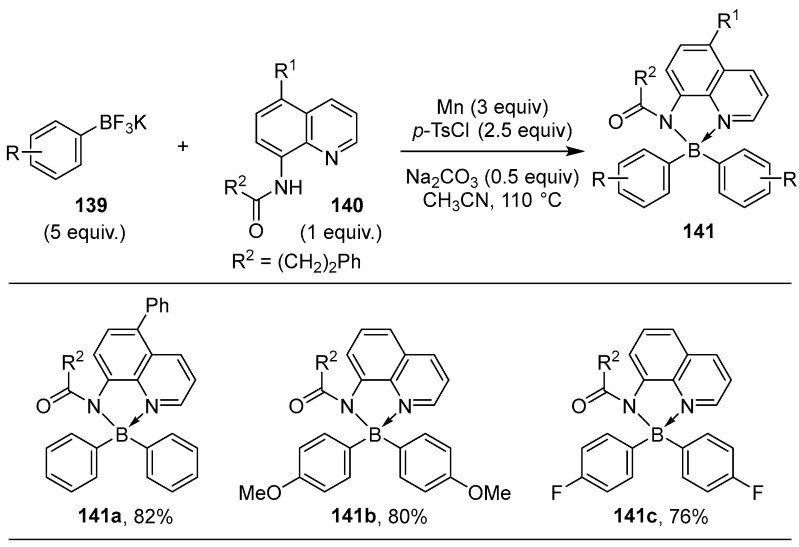
Synthesis of aminoquinolate diarylboron complexes from ArBF_3_K.

A related transformation was recently developed by reacting stable potassium aryltrifluoroborates **139** with acylated 8-aminoquinolines **140**, in the presence of *p*-tosyl chloride, manganese and sodium carbonate (Scheme 45) [94]. Since the aim of the study was to use these compounds as photoredox catalysts, the reaction was depicted on seven substrates. Nevertheless, yields ranging from 76% to 95% were obtained, with a good tolerance for *p*-methoxy (**141b**) and *p*-fluoro (**141c**) substituents.

**Scheme 46:**
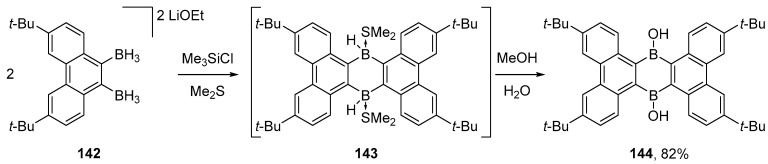
Synthesis of diborinic acid **144** from arylborohydride **142**.

Within the framework of the synthesis of boron-doped anthracenes and pentacenes, Wagner and his coworkers disclosed the dimerization of aromatic 1,2-borohydrides **142** prepared by the reduction of the corresponding BPin adduct with LiAlH_4_. Hydride abstraction by Me_3_SiCl in dimethyl sulfide resulted in the cyclocondensed product **143** (Scheme 46) [95,96,97]. Subsequent hydrolysis afforded diborinic acid **144** with a good 82% yield over two steps.

**Scheme 47:**
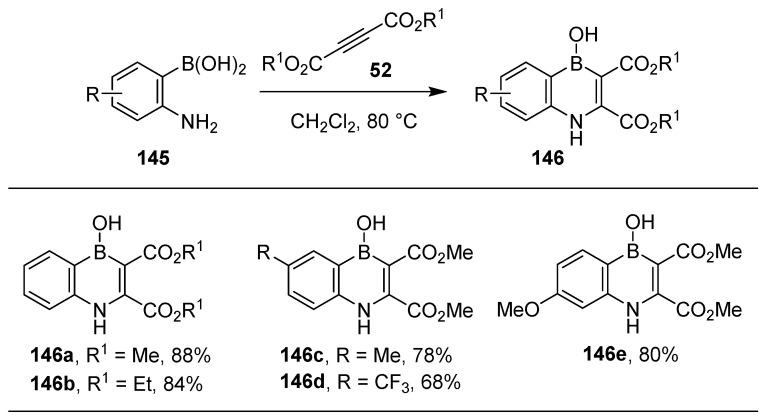
Synthesis of benzofused azaborinols **146**.

2-Amino phenylboronic acids were used in an interesting intermolecular annulation with electron-deficient alkynes (Scheme 47) [98]. The reaction was conducted in CH_2_Cl_2_ in a sealed tube, either at 80 °C or 110 °C under microwave irradiation. It appeared that a high temperature was crucial to achieve the cyclisation and reach good conversions, without evidence of product degradation. Thus, excellent yields were generally obtained with dimethyl- or diethyl-substituted acetylene dicarboxylates (**146a–b**), as well as with electron rich (**146c**, **146e**) or poor (**146d**) phenylboronic acids. The authors demonstrated that borinic acids **146** could be converted into indoles, under palladium catalysis.

**Scheme 48:**
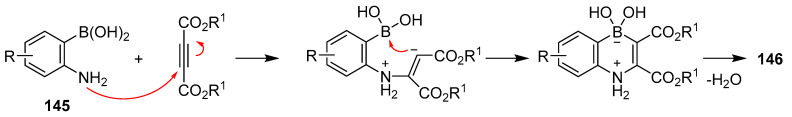
Proposed mechanism for the formation of **146**.

The proposed mechanism implies a two-step process with first a conjugate addition of the aniline onto the triple bond, followed by trapping of the vinyl anion by the boron atom (Scheme 48).

**Scheme 49:**
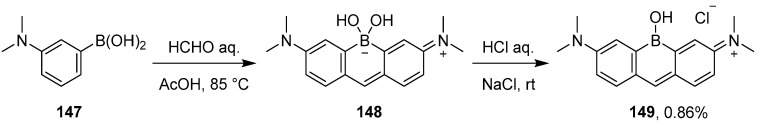
Synthesis of borinate-containing xanthene analog **149**.

The reactivity of boronic acid **147** derived from aniline was illustrated by the synthesis of the red fluorophore **149**, a xanthene analog (Scheme 49) [99]. In this particular example, 3-(*N*,*N’*-dimethylamino)phenylboronic acid **147** was treated with formaldehyde in acetic acid at 85 °C to form borinate **148**. The latter was precipitated with sodium chloride under acidic conditions, leading to borinic acid **149** with a very low yield due to purification issues. It seems that once both aromatic rings have reacted with formaldehyde, the intermediate undergoes cyclization with the concomitant elimination of boric acid, and oxidation.

**Scheme 50:**
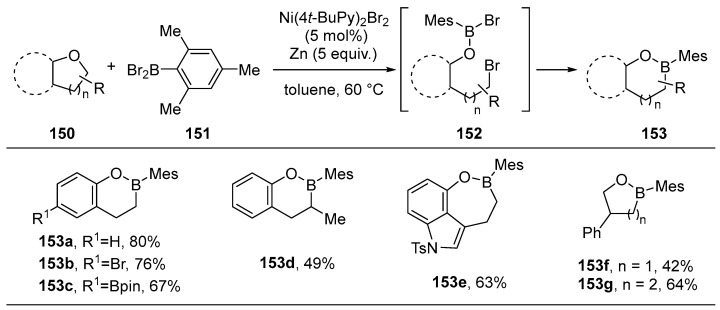
Synthesis of cyclic borinic acids **153** via boron insertion into alkyl ether bond.

Dong et al. described an original synthesis of boron-containing heterocycles by boron insertion of MesBBr_2_ **151** into a series of 2,3-dihydrobenzofurans **150** (Scheme 50) [100]. The reaction is catalyzed by a Ni(II) complex and requires a stoichiometric amount of Zn. Mechanistic investigations, supported by DFT studies, suggest that Zn plays a crucial role in the ring opening to form intermediate **152**. Subsequent rebound is catalyzed by Ni(II), with Zn serving as a reductant. Boron heterocycles **153** that embedded a 5-, 6- or 7-membered ring were smoothly obtained in good to excellent yields.

**Scheme 51:**
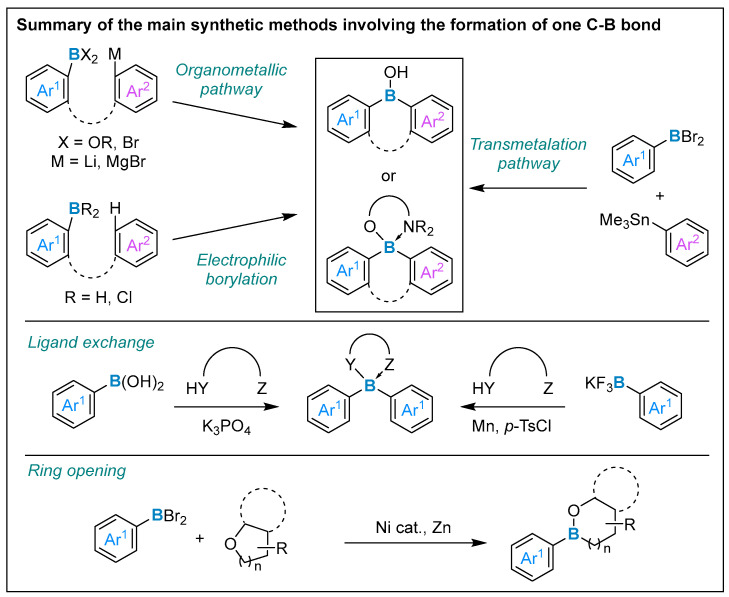
General methods for the synthesis of borinic acid derivatives via one C-B formation.

The main synthetic methods involving the formation of one C-B bond are summarized in Scheme 51. The organometallic pathway has been extensively studied, and offers versatility in terms of molecular diversity (acyclic, cyclic, symmetrical and non-symmetrical) but suffer from a lack of functional compatibility. Other methods are more tolerant toward functionality, but they usually require specific substrates (incorporating a directing group or cyclic ethers, etc.), toxic reagents (tin derivatives), or an excess of organoboron derivatives (ligand exchange).

## 4. Cleavage of One C-B Bond

### 4.1. Synthesis of Four-Coordinated Borinic Acids from Triaryl Boranes

**Scheme 52:**
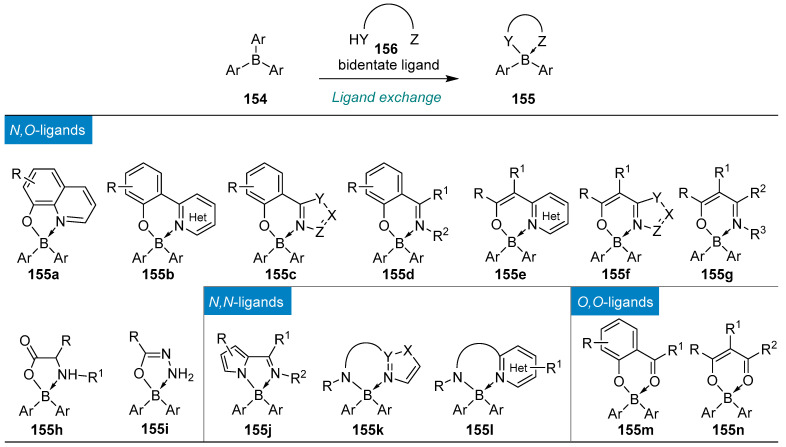
Overview of the bidendate ligands used in the synthesis of diarylborinates **155**.

Four-coordinated diarylborinic acids bearing π-conjugate bidendate ligands have been widely studied for their luminescent properties and applications in optoelectronics (OLED, organic field-effect transistors, imaging materials, etc.) [14]. One of the main and straightforward methods to access such structures relies on the reaction of triarylboranes with a suitable bidentate ligand. Since the reaction proceeds through a protodeboronation process, the presence of an acidic proton on one of the heteroatoms of the ligand is mandatory. Because of this particular mechanism, most of the examples describe the use of triarylboranes bearing three identical aryl groups in order to prevent any mixture of different diarylborinates. As shown in Scheme 52, a large variety of ligands on the organoborinates have been employed and can be sorted into three main categories: *N,O*-ligands [101,102,103,104,105,106,107,108,109,110,111,112,113,114,115,116,117,118,119,120,121,122,123,124,125,126,127,128,129,130,131,132,133,134,135,136], *N*,*N*-ligands [110,137,138,139,140,141,142,143,144,145,146,147,148,149,150,151,152,153,154,155,156,157,158,159,160,161,162,163,164,165,166,167,168] and *O*,*O*-ligands [169,170,171,172,173,174,175,176]. Most of these examples describe the synthesis of diphenylborinates from commercially available triphenylborane.

**Scheme 53:**
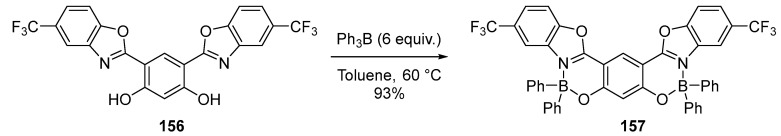
Example of the synthesis of a diborinate complex **157** using Ph_3_B.

A typical example is illustrated in Scheme 53 with the synthesis of the highly fluorescent dyes **157** reported by Ulrich et al. [116]. The borate complex **157** was obtained by reacting 2,5-bis(benzooxazol-20-yl)hydroquinone **156** with six equivalents of triphenylborane in toluene at 60 °C. After recrystallization, pure compound **157** was isolated with a 93% yield.

**Scheme 54:**
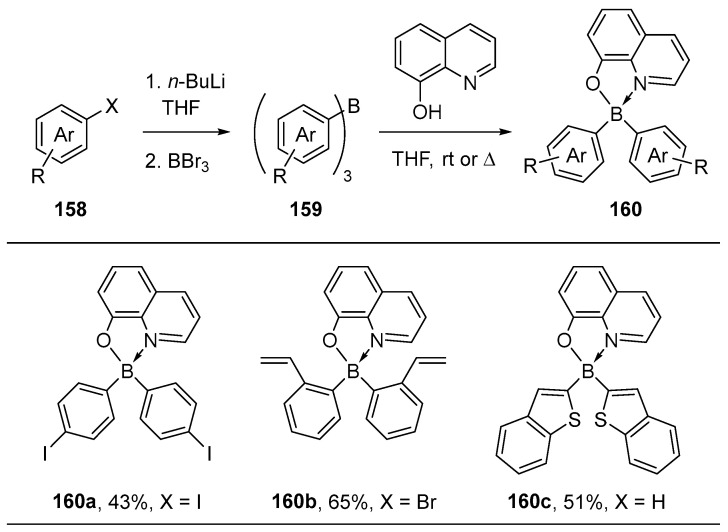
Synthesis of functionalized diarylborinate complexes.

Four-coordinated diarylborinic acids bearing functionalized aromatic moieties or heteroaromatics [103], have also been reported from triarylboranes (Scheme 54). For instance, non-commercially available triarylboranes can be easily prepared by reacting an organolithium species with tribromoborane [101,102,103]. Subsequent treatment of the triarylborane **159** with 8-hydroquinoline in THF afforded **160a–c** in good yields over two steps. Interestingly, compounds **160a–b** are stable enough to be used in post-functionalization processes such as palladium-catalyzed Sonogashira coupling (i.e., **160a**) [102], or ruthenium-catalyzed ring closure metathesis (i.e., **160b**) leading to dibenzoborepines [101].

**Scheme 55:**
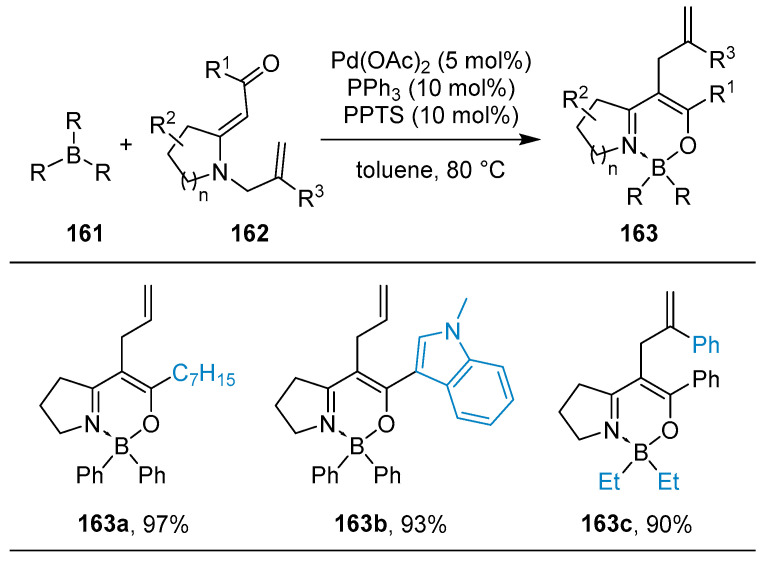
Synthesis of oxazaborinines **163** by Pd-catalyzed rearrangement.

In 2018, Sarpong et al. developed an elegant synthesis of a new class of oxazaborinines, combining Lewis acid activation (R_3_B) with Pd-catalyzed rearrangement [177]. The reaction of triphenyl- or triethylborane **161**, with enaminones **162** under palladium catalysis afforded oxazaborinines **163a–c** in excellent yields, via in situ generation of the *N,O*-ligands (Scheme 55).

**Scheme 56:**
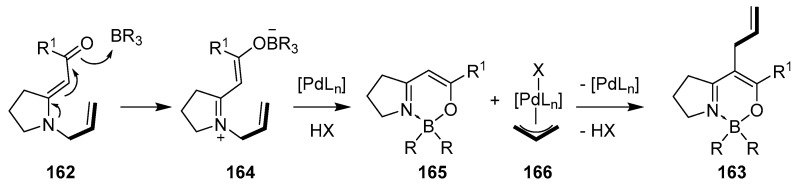
Proposed mechanism for the formation of oxazaborinines **163**.

As outlined in Scheme 56, the reaction proceeds through the formation of iminium **164**, by coordination of the enaminone with borane **161**. Next, a C-N bond activation occurs in the presence of palladium and Brønsted acid catalyst to generate boron enolate **165** and palladium π-allyl complex **166**. Eventually, allylic alkylation of the latter gave rise to the allyl-substituted oxazaborinine **163**.

**Scheme 57:**
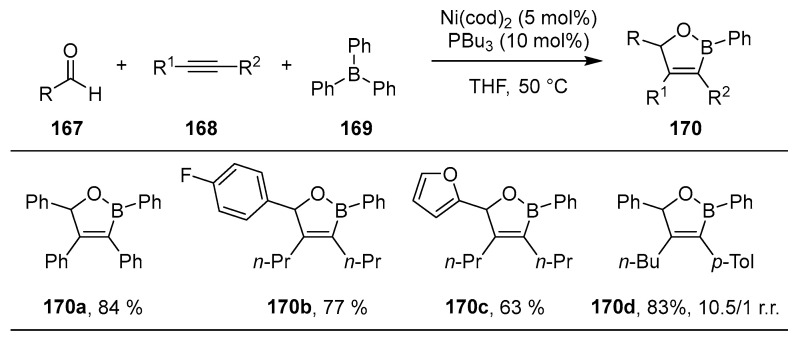
Ni-catalyzed synthesis of oxaboranes **170**.

Stanley et al. reported the direct synthesis of oxaboranes **170**, through a Ni-catalyzed three-component cyclocondensation (Scheme 57) [178]. The versatility of the reaction was illustrated with more than 30 examples, using (hetero)aromatic aldehydes **167**, symmetrical or non-symmetrical alkynes **168**, and triphenylborane **169**. A variety of oxaboranes **170** was isolated in moderate to excellent yields (47–99%), with high regioselectivities (i.e., **170d**, regioisomeric ratio 10.5/1). The reaction proceeds through the formation of an oxanickelacyclopentene by oxidative cyclization with the aldehyde and alkyne, followed by subsequent transmetalation with triphenylborane **169**. These oxaboranes **170** proved to be valuable intermediates for further applications in organic synthesis.

**Scheme 58:**
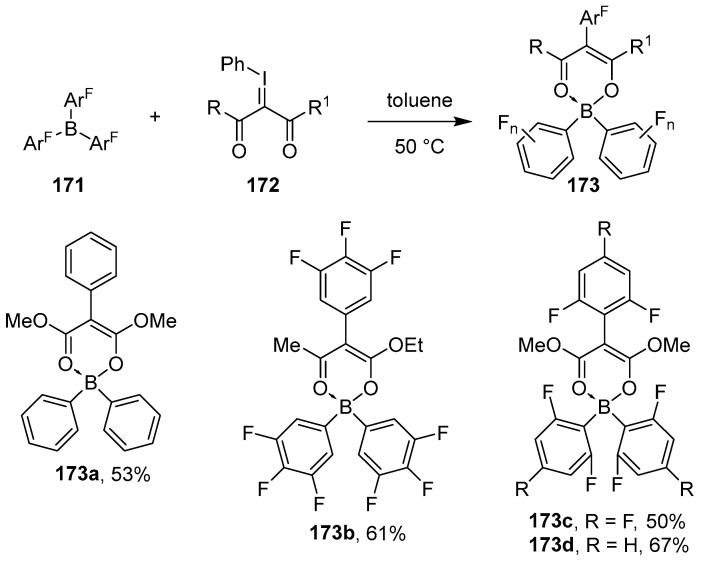
Carboboration of iodonium ylides **172**.

In 2020, Melen et al. described an original access to dienolate-coordinated borinic acids by carboboration of iodonium ylides (Scheme 58) [179]. They showed that mixing triarylboranes **171** with an equimolar amount of acyclic symmetrical or unsymmetrical iodonium ylides in toluene afforded compounds **173a–d** in good yields. DFT calculation revealed that the mechanism proceeds through coordination of the borane to the carbonyls of **172**, followed by boron to carbon aryl migration, and phenyliodine elimination. Overall, the reaction resulted in the 1,3-carboboration of iodonium **172**.

### 4.2. Synthesis of Four-Coordinated Borinic Acids from Tetraarylborate Salts

**Scheme 59:**
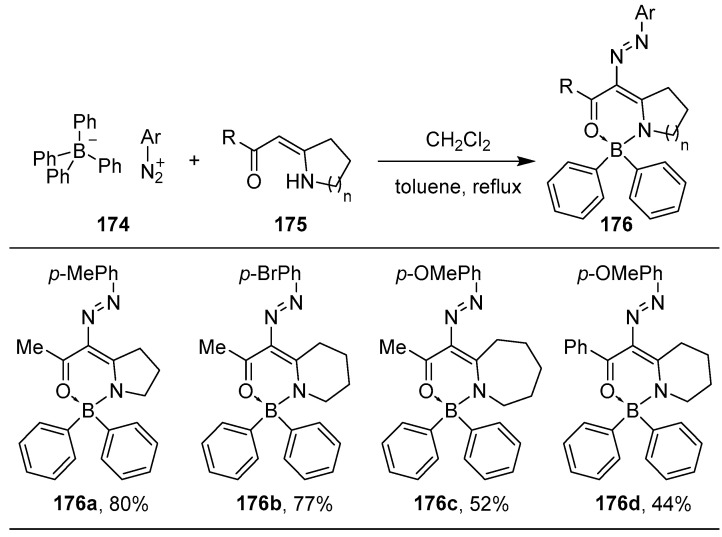
Synthesis of oxazaborinines **176** from benzenediazonium tetraphenylborates **174**.

The synthesis of bicyclic oxazaborinines **176** has been described starting from cyclic β-enaminones **175**, bearing a secondary amino group, and benzenediazonium tetraphenylborates **174** (Scheme 59) [180,181]. The corresponding oxazaborinines **176** were obtained in modest to good yields, depending on the nature of the 4-substituted benzenediazonium and the size of the cyclic enaminones **175**. Some related examples were also described starting from β-enaminoamides [182] and β-enaminonitriles [183].

**Scheme 60:**
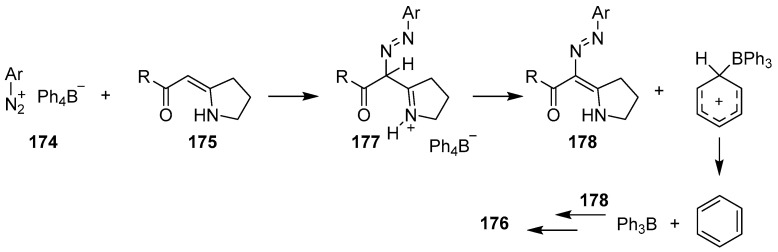
Proposed mechanism for the formation of **176**.

In the proposed mechanism, the initial addition of the enamine **175** onto the diazonium salt **174** leads to the intermediate **177**, which undergoes a protodeboronation process giving rise to triarylborane (Scheme 60) [134]. The latter then reacts with the enaminone **178** to form the oxazaborinine **176**.

**Scheme 61:**
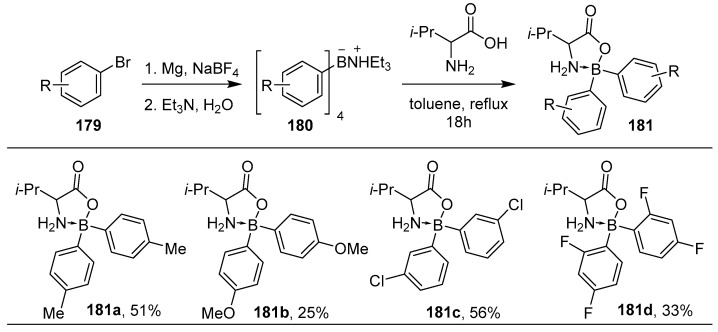
Synthesis of oxazaborolidinones **181**.

An alternative method for the preparation of oxazaborolidinones **181** has been developed by Candeais et al. from tetraarylborates **180**, prepared by the addition of the corresponding Grignard reagents of **179** onto sodium tetrafluoroborate (Scheme 61) [184]. Condensation of the ammonium salts with L-valine in refluxing toluene produced oxazaborolidinones **181**. Although the yields are modest, the reaction tolerates EDG and EWG on the aromatic ring. Some of these compounds showed moderate antitumor activity against colorectal adenocarcinoma cells and human brain astrocytoma cells.

**Scheme 62:**
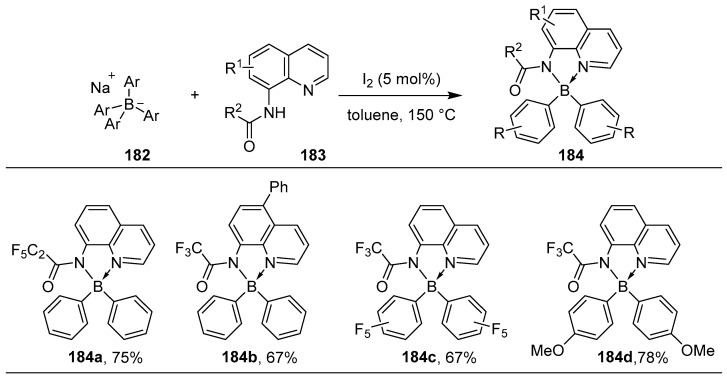
Synthesis of *N,N′*-chelate organoboron aminoquinolates **184** catalyzed by iodine.

Tetraarylborate salts have also been used in the efficient synthesis of fluorescent *N,N*′-chelate organoboron aminoquinolates [185]. It was shown that iodine is able to catalyze the reaction of tetraarylborates **182** with a variety of aminoquinolates **183** in refluxing toluene (Scheme 62). The reaction was exemplified on 20 substrates, with good to excellent yields obtained when R^2^ is an electron-withdrawing group (i.e., CF_3_, C_2_F_5_, **184a–d**). Furthermore, the substitution on the aminoquinolate (i.e., **184b**) and the aryl group (i.e., **184c–d**) is also well tolerated.

**Scheme 63:**
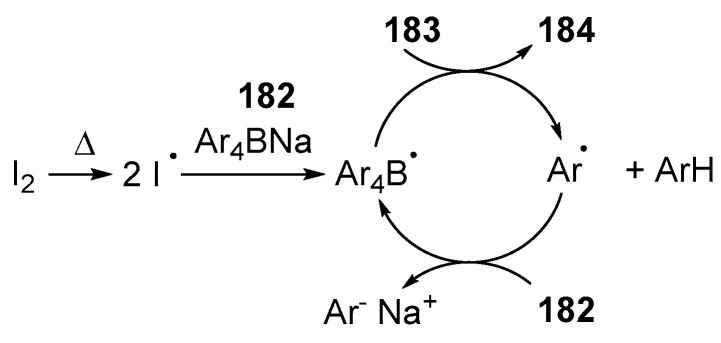
Proposed mechanism for the iodine-catalyzed synthesis of organoborons **184**.

After a series of control experiments, the authors proposed the mechanism depicted in Scheme 63, involving a radical pathway. First, the iodine radical formed by homolytic cleavage, oxidizes Ar_4_BNa **182** to produce the intermediate Ar_4_B^•^ radical. The latter then reacts with the aminoquinoline **183** to give the desired product **184**, ArH and an aryl radical. A final reduction (SET) from **182** to Ar^•^ leads to ArNa and regenerates the Ar_4_B^•^ radical.

### 4.3. Synthesis of Borinic Acids by Hydrolysis or Oxidation of Triaryl Boranes

**Scheme 64:**
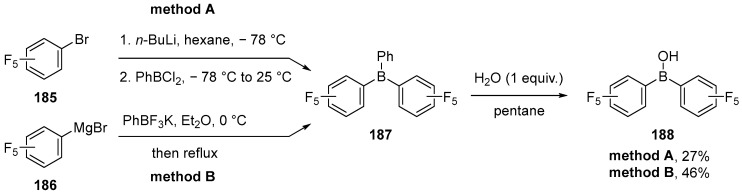
Synthesis of perfluorinated borinic acid **188**.

The hydrolysis and oxidation of triarylboranes has been described in few examples and is limited to specific substrates. For instance, the synthesis of perfluorinated borinic acid **188** was reported following two complementary methods (Scheme 64) [186]. Triarylborane **187** was either synthesized by the addition of Ar^F^Li or Ar^F^MgBr onto PhBCl_2_ or PhBF_3_K, respectively. Selective hydrolysis of **187** with one equivalent of water furnished the corresponding borinic acid **188** with 27% and 46% yields depending on the strategy.

**Scheme 65:**
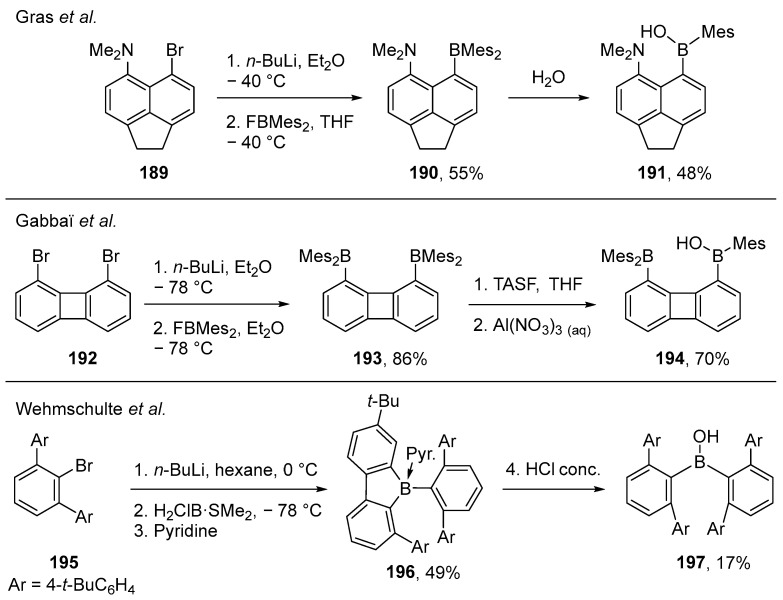
Examples of hydrolysis of triarylboranes [187,188,189].

Other examples are related to the study of the reactivity of sterically hindered triarylboranes towards water (Scheme 65). During their study on frustrated Lewis pairs, Gras and coworkers reported that the crystallization of **190** in heptane led to the formation of borinic acid **191** with a 48% yield, as a result of the partial protodeboronation of **190** [187]. A related reactivity was also observed by Gabbaï et al. with the air-stable bis(dimesitylborane) **193** [188]. Its treatment with TASF (tris(dimethylamino)sulfonium difluorotrimethylsilicate) followed by an aqueous solution of Al(NO_3_)_3_ gave the air- and moisture-stable borinic acid **194** with a 70% yield. It was presumed that the formation of **194** occurred by hydrolysis of the C–B bond promoted by a fluoride ion. A selective C–B bond hydrolysis of 9-borafluorene **196** has also been described by Wehmschulte [189]. They found that prolonged treatment (1 week) of pyridine complex **196** in the presence of concentrated aqueous HCl and air led to sterically hindered diarylborinic acid **197** with a 17% yield after crystallization.

It should be noted that oxidation of triarylboranes into borinic acids has been reported under air or oxygen, but the reactions are limited to specific substrates [57,190]. Furthermore, the oxidation of NaBAr^F^_4_ into the corresponding borinic acid has also been observed (~15% yield) in the presence of an equimolar quantity of ReO_3_Cl complex [191].

## 5. Conclusions

This review summarized the main approaches to access borinic acids, and their chelate derivatives. These methods have been successfully applied to the synthesis of symmetrical and non-symmetrical borinic acids, including boron-containing heterocycles. Many of these organoboron compounds have found applications in organic light emitting diodes (OLEDs), medicinal chemistry, catalysis, material sciences, etc. However, there are still some synthetic challenges that need to be addressed. In particular, most of the syntheses rely on the addition of organometallic species (ArLi, ArMgX) onto electrophilic boron reagents. Although several advances have been made to selectively prepare borinic acids (without the formation of boronic acids and/or triarylboranes), these methods still suffer from a lack of functional group compatibility with the organometallics. Metal-catalyzed strategies have recently emerged to answer this issue but are limited to specific substrates (i.e., cyclic borinic acids), or result in low isolated yields in the case of acyclic borinic acids. The selective synthesis of non-symmetrical borinic acids, especially in the acyclic series, is another major challenge. Except for some isolated examples, there are no functional-group-tolerant and general methods to tackle this issue. The development of such approaches should have a significant impact in the field, allowing the access to new organoboron structures with unexplored properties. Table 1 summarizes the main boron reagents and methods used for the synthesis of borinic acid derivatives, with their advantages and limitations.

## Figures and Tables

**Table 1:** Advantages and limitations of the main boron reagents and methods.

	Boron Reagent	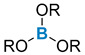	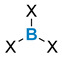	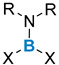	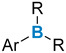	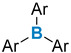
Methods						
Organometallic pathway: poor functional compatibility		+ symmetric/non-symmetric + cyclic/acyclic − moisture sensitive reagents − possible polyaddition of organometallic reagent	+ symmetric + cyclic/acyclic − moisture sensitive reagents − possible polyaddition of organometallic reagent	+ symmetric/non-symmetric + cyclic/acyclic + no polyaddition + catalytic methods exist, tolerant with functional groups − moisture sensitive boron reagents	+ symmetric/non-symmetric + cyclic/acyclic + stable reagent + no polyaddition	
Electrophilic borylation/ Transmetalation: good functional compatibility, no polyaddition to boron reagent			+ symmetric/non-symmetric + cyclic/acyclic − some examples with tin reagents − sometimes requires a chelating group or limited to specific substrates		+ symmetric/non-symmetric + cyclic/acyclic + stable reagent − some examples with tin reagents − sometimes requires a chelating group or limited to specific substrates	
Ligands exchange: good functional compatibility					**ArB(OH)_2_ or ArBF_3_K** + symmetric + acyclic + wide range of ligands + stable reagent − excess reagent	+ symmetric + acyclic + wide range of ligands − usually if Ar ≠ Ph, boron reagents need to be prepared

## Data Availability

Not applicable.
